# ART adherence care strategies among adolescents living with HIV in Ghana's Ashanti Region: a qualitative study

**DOI:** 10.3389/fpubh.2026.1915477

**Published:** 2026-09-16

**Authors:** Doris Hagan, Victor Luckboy Dzramado, Cynthia Yaba Kumah, Charles Domfeh, Joana Edem Koto, Samuel Antwi, Thomas Hormenu

**Affiliations:** 1 Department of Public Health Nursing, School of Nursing and Midwifery, Kwame Nkrumah University of Science and Technology, Kumasi, Ghana; 2 Department of Biostatistics, Cape Coast Teaching Hospital, Cape Coast, Ghana; 3 Department of Midwifery, School of Nursing and Midwifery, Kwame Nkrumah University of Science and Technology, Kumasi, Ghana; 4 Department of Health, Physical Education and Recreation, University of Cape Coast, Cape Coast, Ghana; 5 Department of Biostatistics, Kintampo Health Research Centre, Kintampo-Bono East, Techiman, Ghana; 6 Department of Epidemiology, University of Ghana, Legon, Accra, Ghana

**Keywords:** adolescents living with HIV, antiretroviral therapy adherence, care strategies, counseling, Ghana, implementation, qualitative research, stigma

## Abstract

Adolescents living with HIV (ALHIV) in sub-Saharan Africa carry a disproportionate share of the regional epidemic and consistently record poorer adherence to antiretroviral therapy (ART) than adults. While the statistical correlates of non-adherence are well documented, the everyday strategies adolescents devise to keep themselves on treatment remain comparatively understudied in Ghana. This study explored the ART adherence care strategies of ALHIV in the Ashanti Region, with a view to informing implementable, adolescent-responsive service models. This was the qualitative strand of a convergent parallel mixed-methods study whose quantitative strand surveyed a census of 515 adolescents and recorded 92% self-reported adherence. Twenty-four adolescents aged 14–19 years, all with perinatally acquired infection and all of whom had received documented full disclosure of their HIV status before recruitment, took part in three mixed-sex focus group discussions of eight participants each, one at each of a teaching hospital, a regional hospital, and a district hospital. Discussions lasted approximately 60 min, were conducted in Asante Twi without caregivers present, audio-recorded with consent, transcribed verbatim, independently translated into English with verification against the recordings, and analyzed using inductive thematic analysis in NVivo with independent peer coding and member checking. The Information-Motivation-Behavioral Skills (IMB) model framed the interpretation. Four themes were generated: ART reminder strategies, comprising social support, routine activity cues, and mobile phone or watch alarms; stigma and concerns about privacy, comprising concealment practices and unease about HIV public education campaigns that display ART packaging; emotional and psychological impact, centring on acceptance and coping through counseling; and motivation and commitment to treatment, comprising personal goals and a desire for good health. Adolescents combined family reminders, daily routines, and concealed pill containers to protect both adherence and anonymity, while counseling and future aspirations sustained long-term commitment. Ghanaian adolescents are active managers of their own treatment rather than passive recipients of care. Service delivery should formalize the informal strategies adolescents already use, strengthen confidential reminder and counseling supports, and revise public messaging so that awareness campaigns do not inadvertently disclose status.

## 1 Introduction

Adolescence is a formative period of identity formation, social experimentation, and growing autonomy, and these same developmental features make medication-taking unusually difficult for young people living with a chronic, stigmatized condition. Globally, adolescents remain among the populations least well served by the HIV response, and sub-Saharan Africa continues to host the overwhelming majority of adolescents living with HIV (ALHIV) (1, 2). The demographic weight of this group is considerable, since the region is undergoing an epidemiological and demographic transition that places an expanding cohort of young people at the center of unfinished HIV control efforts (3). For adolescents, the central clinical task is not only to initiate antiretroviral therapy (ART) but to sustain near-perfect daily adherence over many years, because durable viral suppression depends on it.

The evidence consistently shows that ALHIV adhere less well than adults. A systematic review of adherence among HIV-positive adolescents in sub-Saharan Africa identified a dense web of individual, social, and structural barriers that interact to undermine consistent pill-taking (4). A mixed-methods systematic review and meta-analysis of 66 studies of adolescents aged 10–19 years in the region likewise concluded that adherence remains low despite numerous interventions, and that the principal barriers cluster into social, patient-based, economic, health-system, therapy-based, and cultural domains, while the main facilitators are social support, counseling, and ART education combined with confidentiality (4). An earlier meta-analysis reported that adherence falls measurably as young people move through adolescence, underscoring the developmental specificity of the problem (5).

In Ghana, the picture is broadly similar. National estimates indicate that 334,721 people were living with HIV in 2024, when the country recorded 15,290 new infections, approximately 31% of which occurred among young people aged 15–24 years and 1,823 of which, representing 11.9%, occurred among adolescents aged 10–19 years (6). A nationwide systematic review and meta-analysis of 14 studies covering 4,436 participants estimated pooled optimal ART adherence at 70% (95% CI 58 to 81), well below the level required for reliable viral suppression, with adolescents and young adults adhering at a still lower pooled rate of 66% (95% CI 46–84) (7). Facility-based and qualitative work from Ghana has documented how disclosure difficulties, transport and food insecurity, school environments, and fear of being identified shape the adherence experiences of young people (8, 9). Studies of Ghanaian adolescents with perinatally acquired infection have further shown that the burden falls not only on physical health but on quality of life and psychosocial wellbeing (10), and mixed-method work at public facilities has highlighted how both patient-level and service-level factors operate together (11, 12). What this literature establishes is the architecture of the problem. What it describes less fully, particularly for Ghana, is the inventory of practical, self-generated strategies that adolescents themselves use day to day to stay on treatment in the face of these barriers.

This study is framed by the Information-Motivation-Behavioral Skills (IMB) model, which holds that adherence-related information and adherence-related motivation work largely through behavioral skills to produce adherence behavior (13, 14). The model has been applied productively to youth newly initiating ART and offers a useful lens for interpreting how adolescents translate knowledge and motivation into concrete routines (15). Importantly, most adherence-support interventions evaluated in low- and middle-income settings have been designed and tested by researchers and programme planners, with comparatively little attention to the behavioral skills adolescents have already engineered for themselves (16, 17). Surfacing those existing skills is valuable for implementation, because strategies that young people have already adopted are, by definition, acceptable and feasible within their own contexts, and they can be formalized and scaled with relatively low friction. This orientation aligns with the broader policy ambition of reaching the 95–95–95 targets, which cannot be met without sustained adherence among the youngest people in care (18).

Qualitative inquiry is well suited to this task because it privileges the meaning that adolescents attach to their condition and treatment, and because group discussion can elicit the shared, normative practices that individuals adopt within a community of peers (19). Existing qualitative studies from the region have richly described barriers such as fear of unintended disclosure and difficult transitions to self-management (20), but the affirmative, capability-oriented question of how adolescents successfully care for themselves has received less systematic attention in Ghana. Addressing that gap, the present study explored the ART adherence care strategies of ALHIV in the Ashanti Region of Ghana. The specific objective was to identify and interpret the care strategies adolescents use to enhance adherence, so that routine services can build on, rather than duplicate, what young people are already doing. By centring adolescent agency and locating the findings within an implementation frame, the study aims to provide service-relevant evidence for adolescent-friendly ART programmes in Ghana and comparable settings.

## 2 Materials and methods

### 2.1 Study design and relationship to the parent mixed-methods study

This article reports the qualitative strand of a larger convergent parallel mixed-methods study of the determinants of ART adherence among ALHIV in the Ashanti Region of Ghana. In a convergent parallel design, quantitative and qualitative data are collected during the same phase, analyzed separately, and then merged so that statistical relationships can be explained and enriched by participants' accounts (21).

The quantitative strand administered a 48-item adapted questionnaire, incorporating items from the AIDS Clinical Trials Group and LifeWindows IMB adherence instruments, to a census of 515 adolescents aged 10–19 years (mean age 15.44 years, SD 2.62; 49.5% male, 50.5% female) enrolled at the same three ART sites. It recorded a high level of self-reported adherence at 92%, a weak and non-significant negative correlation between adherence rate and viral load (*r*_*s*_ = −0.052, *p* = 0.235), and several significant predictors of adherence. These included knowledge of potential ART side effects (B = −5.484, β = −0.145, 95% CI−8.904 to−2.064), knowledge of medication interactions with alcohol and other drugs (B = 5.604, β = 0.114, 95% CI 0.894–10.314), higher educational level (B = −5.076, β = −0.264, 95% CI−7.581 to −2.571), and better economic status (B = 4.025, β = 0.114, 95% CI 0.975–7.075). The quantitative findings are reported separately and are not duplicated here.

The two strands answer complementary questions. The quantitative strand established *how much* adherence occurred and *which measurable factors* predicted it, but its regression models explained only a modest share of variance and could not describe the mechanisms through which adolescents actually achieved the observed 92% adherence. The qualitative strand reported here addresses that mechanistic gap by documenting the concrete practices adolescents use. The strands are brought together in section 4.6, where the qualitative themes are used to interpret the quantitative results. This article presents the qualitative strand alone because the care strategies it describes constitute a self-contained, implementation-relevant contribution whose depth would be lost if compressed into a combined report, and because the intended readership for service-design evidence differs from that for the epidemiological analysis.

The qualitative strand used a descriptive phenomenological orientation operationalised through focus group discussions (FGDs), chosen to capture the social and cultural context in which adolescents manage their treatment and to allow participants to build on one another's experiences. Reporting follows the Consolidated Criteria for Reporting Qualitative Research (COREQ) (22).

### 2.2 Study setting

The study was conducted in the Ashanti Region, which lies in the middle belt of Ghana and is the country's most populated region, with an estimated 5,432,485 residents and the largest adolescent population of any region, about 1,353,768 young people aged 10–19 years (23). The region is served by approximately 1,654 health facilities across the levels of care, but only a subset provides comprehensive ART services for adolescents. Data collection took place over 4 months at three facilities offering adolescent ART services: one teaching hospital, one regional hospital, and one district hospital. These sites were selected purposively because they function as referral centers with well-established adolescent ART services, serve diverse patient populations drawn from across the region, and manage comparatively large numbers of ALHIV, thereby offering rich and varied accounts relevant to the study objective.

### 2.3 Theoretical framework

The study was guided by the IMB model of ART adherence, which posits that adherence information and adherence motivation operate, in large part, through adherence-related behavioral skills to influence adherence behavior (13, 14). The conceptual basis used to organize the constructs is depicted in Figure 1: Conceptual Base of ART Adherence among ALHIV. Within the qualitative analysis, the model functioned as a sensitizing framework that helped locate adolescents' described strategies within the domains of information, motivation, and behavioral skills, without constraining the inductive generation of themes. The explicit mapping of the generated themes onto the model's components is presented in section 4.1.

**Figure 1:**
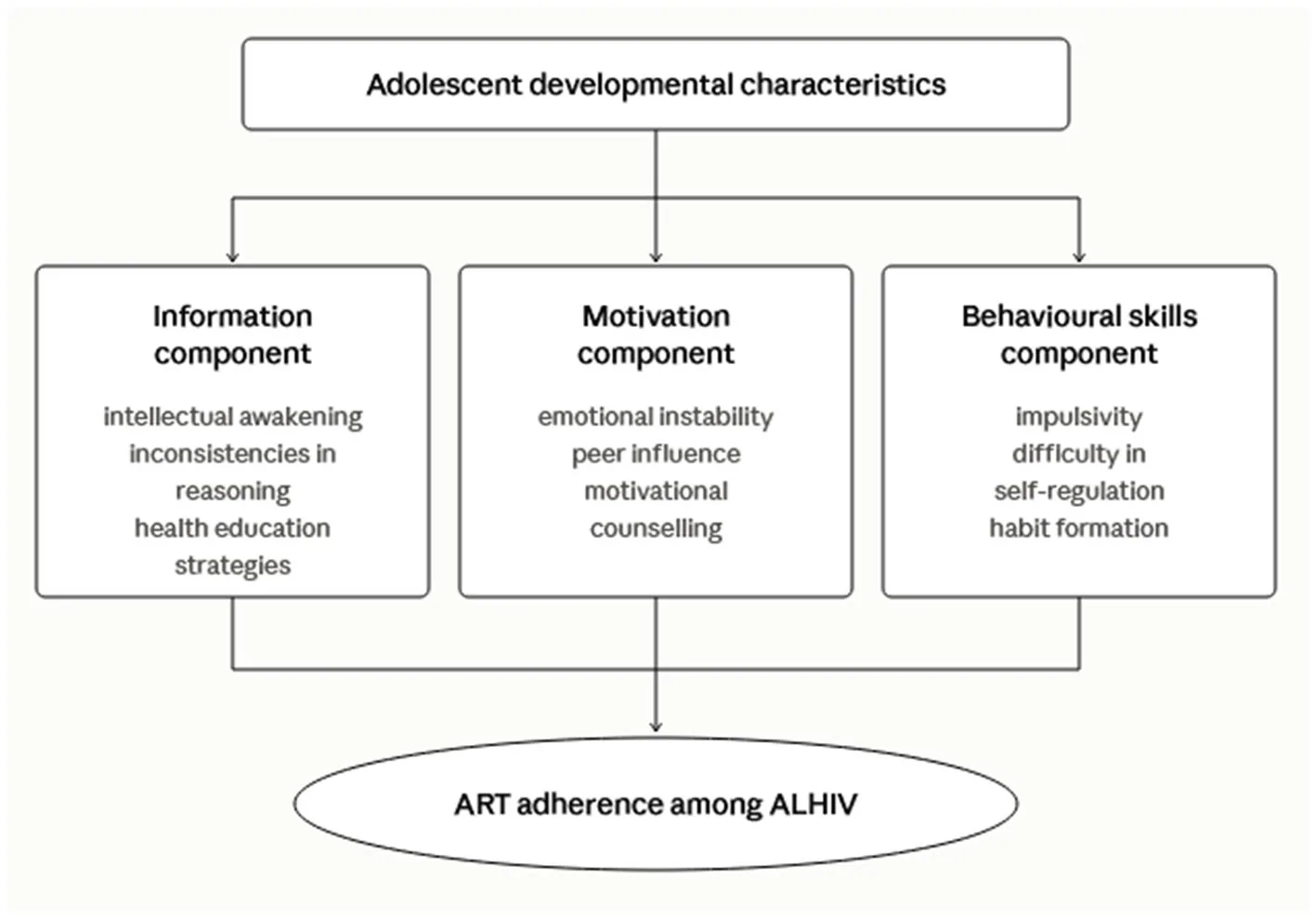
Conceptual Base of ART Adherence among ALHIV.

### 2.4 Study population, eligibility, and sampling

The qualitative population of interest comprised adolescents living with HIV who were enrolled in ART care at the selected facilities. Eligibility for the FGDs required that the adolescent was aged **14 to 19 years**, had been receiving ART at the facility, had received documented full disclosure of their HIV-positive status, and was judged competent to provide informed assent or consent and to engage meaningfully in a group discussion of a sensitive subject.

This age band is narrower at its lower bound than the 10–19 year range used in the quantitative strand, and the restriction was deliberate. Older adolescents were prioritized in selection for three reasons. They were cognitively and emotionally more mature and therefore better able to engage with the abstract and sensitive material under discussion. They had generally been on ART for longer and had encountered a wider range of adherence challenges, including the transition to boarding school and periods of independent medication management. And they were better able to understand the study and to assent to participation with genuine comprehension, which is ethically important where the group setting itself carries disclosure implications. Adolescents in early adolescence, aged 10 to 13 years, were therefore not eligible for the FGDs, although they were included in the quantitative census. Three participants were aged 14 at the time of the discussions and had all been living with a known diagnosis since childhood; their inclusion is considered in the reflexive account at section 2.9 and among the limitations at section 4.8.

For this strand, 24 adolescents were purposively selected and organized into three focus groups of eight participants each, with one group convened at each facility. Purposive selection sought variation in sex, age within the eligible band, level and type of schooling including boarding and day attendance, household composition, and ART adherence experience, so that the groups would surface a range of practices rather than a single normative account. Group composition of eight balanced the need for manageable individual contributions against a group size large enough to generate dynamic exchange.

### 2.5 Verification of disclosure status

Because a substantial proportion of perinatally infected adolescents in sub-Saharan Africa have not received full disclosure of their status even in mid-adolescence, we treated confirmed disclosure as an explicit eligibility requirement rather than an assumption. Evidence from a closed cohort of 813 perinatally infected adolescents in the Eastern Cape of South Africa found that the proportion aware of their HIV-positive status rose from 63.1% at the first round to 85.5% by the third, with older age (adjusted odds ratio 1.27, 95% CI 1.08–1.48) and urban residence (aOR 2.85, 95% CI 1.72–4.73) predicting disclosure (24). A systematic review of pediatric disclosure in resource-limited settings similarly documents wide variation in practice and timing (25). Given that every participant in this study had acquired HIV perinatally, this consideration was directly material to recruitment.

Disclosure status was verified in three sequential steps before any adolescent was approached. First, the ART clinic nurse responsible for each adolescent's care reviewed the clinical record and confirmed that full disclosure had been documented by the facility's counseling team, together with the approximate age at which disclosure occurred. Second, the caregiver was asked, during the separate consent conversation, to confirm that the adolescent knew their status. Third, at the start of the individual pre-FGD briefing, the researcher confirmed with the adolescent directly, in general and non-leading terms, that they were aware of the condition for which they were receiving treatment and were willing to discuss it in a group of peers who were also receiving the same treatment. Any adolescent for whom disclosure could not be confirmed at all three steps was not enrolled in the qualitative strand. This procedure protected against the serious harm of inadvertent disclosure occurring within a group setting, and it means that the accounts reported here come exclusively from adolescents with established awareness of their status, in most cases of several years' standing.

### 2.6 Data collection

A semi-structured FGD guide was developed from established qualitative focus group methodology and refined to elicit detailed, practical accounts of adolescents' care practices (26, 27). The guide invited participants to describe how they take and store their medication, how they remember doses, how they protect themselves from opportunistic infection, the difficulties they encounter, and what sustains their adherence. It opened with a welcome and ground rules, moved through open-ended questions and probes, and closed with an exit question. The guide was piloted with a comparable group of adolescents at Goaso Government Hospital in the Ahafo Region who did not take part in the main study, and was refined for clarity and contextual relevance.

Four registered nurses with prior experience in health-related research and familiarity with HIV service settings were recruited and trained over 2 days to support data collection. Training covered the study objectives, the FGD guide, techniques for maintaining neutrality, procedures for obtaining informed consent and assent, confidentiality, and the sensitive handling of HIV-related and treatment-related topics.

Each of the three focus groups was **mixed-sex**, comprising both male and female participants, on the reasoning that the care strategies under discussion were not sex-specific and that mixed composition would surface a fuller range of practices. Participants within each group were drawn from the same ART clinic and therefore **may have encountered one another at routine clinic appointments**, though they were not recruited as pre-existing social groups and no adolescent was asked to disclose any prior acquaintance. Because all participants at a given site were already attending the same adolescent ART service, group membership did not itself reveal any information that was not already apparent from clinic attendance.

**Caregivers were not present during recruitment conversations with adolescents, during the pre-FGD briefing, or during the discussions themselves**. Caregiver consent was obtained in a physically separate conversation, so that the adolescent's own decision to assent was made without a caregiver observing. Each FGD was facilitated by the lead researcher with one trained assistant acting as note taker, and no clinical staff responsible for the participants' care were present in the room.

Discussions were conducted in **Asante Twi**, the language spoken in the region, to allow participants to express themselves freely, and each session **lasted approximately 60 min**. Sessions were held in a hospital conference room behind closed doors, at times scheduled for the participants' convenience and generally aligned with existing clinic appointments to avoid additional travel cost. Each participant was assigned a number and asked to state it before speaking, so that contributions could be tracked without using names. Sessions were audio-recorded with participants' consent, and a reflective file was maintained to document non-verbal cues. Rapport-building preceded each session. No financial incentive was offered, although refreshments were provided afterwards, and a debriefing session followed each discussion.

### 2.7 Language, transcription, and translation

Audio recordings in Asante Twi were transcribed verbatim in the original language by a bilingual transcriber fluent in Asante Twi and English. The Twi transcripts were then translated into English by a second bilingual member of the research team who had not performed the transcription. To verify translation fidelity, a third bilingual team member independently reviewed each English transcript against the original audio recording, with particular attention to idiomatic expressions, euphemisms used for HIV and for medication, and terms carrying stigma connotations that do not translate directly. Discrepancies were resolved by discussion among the three, with the original audio treated as the authoritative source. Formal back-translation into Twi was not performed; instead, the audio-to-translation verification described above was used, and this is acknowledged as a methodological limitation in section 4.8. Participants' own phrasing was preserved wherever an English equivalent existed, and quotations presented in the Results have been edited only to remove filler words and to expand abbreviations for readability.

### 2.8 Data analysis and determination of saturation

Transcripts were checked for accuracy against the recordings. Analysis followed an inductive thematic approach, which supports the identification of patterned meaning across accounts and accommodates both explicit and implicit content (28). The lead analyst read the transcripts repeatedly to become immersed in the data, noting recurring phrases related to care strategies. Transcripts were imported into NVivo, and meaningful segments were coded against a shared codebook to standardize the process. A second team member experienced in NVivo served as an independent peer coder, and discrepancies were resolved through discussion until consensus codes were agreed. Codes were grouped into candidate themes, which were refined iteratively and interpreted in relation to the research objective.

Saturation was assessed prospectively rather than asserted retrospectively, following the distinction between code saturation, the point at which no new codes emerge, and meaning saturation, the point at which existing codes are fully understood (29). The three groups were stratified by facility level, with one group per stratum, an approach for which one group per stratum has been shown sufficient to identify the range of issues present, while two per stratum yield a more comprehensive understanding (29). Operationally, the team coded each transcript before conducting the subsequent discussion and maintained a running code inventory. All codes underpinning the four themes reported here had appeared by the end of the second group. The third discussion generated no new codes and instead added contextual depth to codes already established, notably around boarding-school concealment and treatment interruption during travel. On this basis the team judged that code saturation had been reached, while noting in section 4.8 that with only one group per stratum, meaning saturation for facility-specific issues is less securely established. Documentation was kept in the codebook to support transparency, and the qualitative findings were subsequently triangulated with the quantitative strand. The de-identification protocol, the full codebook, the de-identified excerpts mapped to codes, and the coded thematic summaries are provided in the Supplementary Material.

### 2.9 Reflexivity and researcher positionality

Reflexivity is treated here as a continuous and collaborative practice of examining how the research team's subjectivity and position shaped the process, rather than as a one-off disclosure (30).

The lead researcher is a Ghanaian female nurse and doctoral candidate in health promotion, and the data collection assistants were registered nurses. This positionality carried a specific and consequential risk in this study. The research team wore nursing uniforms during data collection and were introduced to participants by facility heads as part of the health team. This decision was originally made to put adolescents at ease in a familiar clinical environment, and participants did appear more relaxed as a result. On reflection, however, we recognize that the same uniform and the same introduction may have positioned the team as clinical authority figures rather than as independent researchers. Adolescents who associate nurses with the dispensing of their medication and the monitoring of their adherence may have felt an implicit pressure to present themselves as adherent, and may have been reluctant to report missed doses to someone they perceived as part of their care team. This is a plausible source of the strongly adherence-affirming tone of many accounts.

Several steps were taken to mitigate this dynamic, though we do not claim they eliminated it. Facilitators opened each session by stating that everyone finds treatment difficult at times, explicitly normalizing non-adherence before any question was asked. Facilitators were trained to respond without judgement to reports of missed doses, and did so; several participants went on to describe missed doses, concealment-driven omissions, and in one case a treatment interruption lasting about a year, which suggests the normalizing strategy had at least partial effect. Participants were told that their responses would not be entered into their clinical records and would not affect the care they received, and no clinician responsible for their treatment was present. Nevertheless, the possibility that the accounts skew toward adherence remains, and it is treated as a limitation rather than as a resolved problem.

A second reflexive consideration concerns the suitability of group discussion for this population and the position of the youngest participants within it. All participants had perinatally acquired infection, and adolescents in this situation are frequently reluctant to speak about their status in front of others. Younger adolescents in particular may feel compelled to participate by the perceived authority of researchers, providers, or accompanying caregivers. Three participants in this study were aged 14, and we gave specific attention to their position. Consistent with the ethical protocol, their agreement was recorded as **written assent** rather than verbal assent alone, alongside separately obtained written parental consent, and the assent document was read aloud to them in Twi with an opportunity to ask questions before signing. Their caregivers were not present when assent was sought or during the discussion itself. Facilitators monitored participation across the group and did not direct questions at quieter members in a way that would oblige them to speak. Even with these safeguards, we accept that a 14-year-old in a group containing 18- and 19-year-olds may defer to older participants or echo an emerging consensus, and we did not analyse the data in a way that would allow us to detect such deference reliably.

We also recognize the countervailing consideration that a group of peers all receiving the same treatment can reduce isolation and enable disclosure that would not occur in an individual interview, and participants did build on one another's accounts, with several volunteering practices only after hearing a peer describe something similar. Even so, group format is unlikely to be the right vehicle for the most private or non-normative experiences, and section 4.8 discusses what this means for interpreting the findings. The team held debriefing discussions after each session in which these dynamics were reviewed, and the reflective file informed both coding and interpretation.

### 2.10 Trustworthiness

Trustworthiness was pursued through the criteria of credibility, transferability, dependability, confirmability, and authenticity (31). Credibility was supported by prolonged engagement, attention to non-verbal cues, triangulation across data sources and across the two strands of the parent study, member checking with FGD participants, and peer debriefing with two qualitative research experts. Dependability was supported by an audit trail covering the interview guide, coding decisions, and peer review for consistency. Confirmability was supported by the reflective journal maintained throughout data collection and by external auditing of the analysis by the doctoral supervisors. Transferability was supported by thick description of the context and participants, including the full participant characteristics presented in Table 1. Authenticity was supported by careful, faithful coding of participants' own words and by the presentation of direct quotations attributed to individual participants.

**Table 1:** Characteristics of the 24 adolescents participating in focus group discussions, Ashanti Region, Ghana.

Code	FGD group (facility level)	Sex	Age (years)	Educational level	Boarding or day	Living arrangement	Mode of acquisition	Age at full disclosure
P1	1 (Teaching hospital)	Male	15	JHS 3	Day	Grandmother	Perinatal	Childhood
P2	1	Male	17	SHS 2	Boarding	Relative (orphan)	Perinatal	Early adolescence
P3	1	Female	14	JHS 1	Day	Parents	Perinatal	Childhood
P4	1	Male	17	SHS 2	Boarding	Mother	Perinatal	Early adolescence
P5	1	Male	14	JHS 2	Day	Parents	Perinatal	Childhood
P6	1	Male	17	SHS 3	Boarding	Parents	Perinatal	Early adolescence
P7	1	Female	18	Tertiary year 1	Day	Parents	Perinatal	Adolescence
P8	1	Female	16	SHS 2	Boarding	Parents	Perinatal	Childhood
P9	2 (Regional hospital)	Male	19	Tertiary year 2	Day	Parents	Perinatal	Adolescence (SHS 1)
P10	2	Male	15	SHS 1	Boarding	Parents	Perinatal	Childhood
P11	2	Female	18	SHS 3	Boarding	Parents	Perinatal	Adolescence (SHS 2)
P12	2	Male	18	SHS 3	Day	Parents	Perinatal	Childhood
P13	2	Male	14	JHS 1	Boarding	Mother	Perinatal	Childhood
P14	2	Male	15	JHS 3	Day	Grandmother (orphan)	Perinatal	Childhood
P15	2	Male	16	SHS 1	Boarding	Father	Perinatal	Early childhood (age 3)
P16	2	Female	17	SHS 2	Day	Mother	Perinatal	Childhood
P17	3 (District hospital)	Male	16	SHS 1	Boarding	Parents	Perinatal	Childhood
P18	3	Female	15	SHS 1	Boarding	Mother	Perinatal	Early childhood
P19	3	Male	18	SHS 3	Day	Father	Perinatal	Childhood
P20	3	Female	19	Tertiary year 2	Boarding	Parents	Perinatal	Early adolescence
P21	3	Male	17	SHS 1	Day	Mother	Perinatal	Adolescence
P22	3	Female	18	Tertiary year 1	Day	Mother	Perinatal	Childhood
P23	3	Male	16	SHS 1	Boarding	Parents	Perinatal	Early adolescence
P24	3	Female	18	SHS 3	Boarding	Father	Perinatal	Early adolescence

JHS, junior high school; SHS, senior high school. Participant codes are pseudonymous and do not correspond to any clinic identifier.

### 2.11 Ethics statement

The studies involving human participants were reviewed and approved by the Ghana Health Service Ethics Review Committee (GHS-ERC:004/04/23) and the Komfo Anokye Teaching Hospital Institutional Review Board (KATH IRB/CA/093/23), with an introductory letter from the Department of Health, Physical Education and Recreation, University of Cape Coast. Permission was also obtained from the Ashanti Regional Directorate of the Ghana Health Service, the relevant district health directorates, and the three participating facilities.

Written informed consent was obtained from participants aged 18 years and above. For participants below 18 years, written assent was obtained from the adolescent together with written informed consent from a parent or guardian, sought in a separate conversation as described in section 2.6. Consent and assent documents were provided in English or Twi and read aloud. Before participation, adolescents and their caregivers were fully informed of the study's objectives, procedures, and possible risks, including the possibility of distressing emotions. Participants were free to decline to share anything they preferred to keep private and to withdraw at any time without any effect on the services they received. Discussions were conducted in private, transcripts were identified only by codes, neutral and non-stigmatizing language was used throughout, and debriefing sessions followed each FGD to address any emotional concerns, with onward referral to facility counseling available if needed. The study was conducted in accordance with the journal's bioethics requirements and the principles of the Declaration of Helsinki.

## 3 Results

### 3.1 Participant characteristics

Twenty-four adolescents took part across three focus groups of eight participants each, one at each participating facility. Participants were aged 14–19 years, with the sample weighted toward older adolescents: three were aged 14, four were 15, four were 16, five were 17, six were 18, and two were 19. Fifteen participants were male and nine were female. Thirteen participants were in junior or senior high school boarding accommodation at the time of the discussions and eleven attended as day students, an eleven to thirteen split that proved analytically important because schooling arrangement shaped both the concealment pressures adolescents faced and the reminder strategies available to them. Educational level ranged from junior high school year one to the second year of tertiary study, with five participants in junior high school, fifteen in senior high school, and four in tertiary education. Participant characteristics are presented in Table 1.

As Table 1 shows, **all 24 participants had perinatally acquired infection**, which situates this sample squarely within the population whose transition through adolescence carries the distinctive challenges of lifelong treatment begun before memory and of a diagnosis disclosed by others rather than discovered. Age at full disclosure varied: fourteen participants had learned their status during childhood or early childhood, six during early adolescence, and four during adolescence proper, with several able to date the moment to a specific school year. One participant had been told at approximately 3 years of age. This variation is consequential for the emotional trajectories described in Theme Three, since those disclosed to in adolescence recounted a sharper and more recent adjustment than those who had grown up with the knowledge.

Household composition also varied in ways that bear directly on the reminder strategies reported in Theme One. Twelve participants lived with both parents, six with their mother alone, three with their father alone, two with a grandmother, and one with another relative. Two participants described themselves as orphaned and dependent on a grandmother or other relative, and both linked their adherence explicitly to that relationship or to their awareness of its fragility. Adolescents living with a grandparent drew on different reminder resources than those living with a parent, boarding students faced surveillance pressures that day students did not, and those in economically precarious households described food insecurity interacting directly with a regimen requiring doses to be taken with meals. The variation deliberately sought at sampling is therefore visible in the accounts that follow.

### 3.2 Overview of themes

Through inductive thematic analysis, four themes were generated: ART reminder strategies, stigma and concerns about privacy, the emotional and psychological impact of HIV, and motivation and commitment to treatment. Each theme contained sub-themes that together describe how adolescents organize their daily treatment and protect their wellbeing. The full thematic structure is presented in Table 2.

**Table 2:** Themes and sub-themes generated from focus group discussions with adolescents living with HIV in the Ashanti Region.

Theme	Sub-themes
Theme One: ART reminder strategies	Social support; Activity reminders; Technology (phone or watch alarm)
Theme Two: Stigma and concerns about privacy	Maintaining privacy regarding medication; Concerns about public education campaigns
Theme Three: Emotional and psychological impact	Acceptance and coping through counseling; Residual and unresolved concerns
Theme Four: Motivation and commitment to treatment	Personal goals and ambitions; Desire for good health

As Table 2 indicates, the four themes move from the concrete mechanics of remembering and taking medication, through the social risks that shape how adolescents handle their treatment, to the inner emotional work of acceptance, and finally to the forward-looking motivations that anchor long-term commitment. Read together, the themes show adolescents acting as deliberate managers of their own care rather than passive recipients of instruction. The sections that follow present each theme in turn, each accompanied by a table of representative quotations drawn from the discussions and attributed to individual participants whose characteristics are given in Table 1.

### 3.3 Theme one: ART reminder strategies

At the time of the discussions, each adolescent knew why they were taking ART, and most understood how important consistent adherence was. From their narratives, participants described a repertoire of strategies for remembering to take medication, falling into three sub-themes: social support, activity reminders, and technology. Representative quotations are presented in Table 3.

**Table 3:** Representative quotations for Theme One: ART reminder strategies.

Sub-theme	Representative quotations
Underlying concern	“The nurses have been telling me that taking my medications on time is important, but I sometimes forget if I am too busy with school activities. I needed something that will remind me every day.” (P17, male, 16 years, SHS 1)
	“I used to not take my medicine in the beginning because of hunger and forgetfulness. But for now, I have set an alarm for my medication. So immediately the alarm goes off I would have to take my medicine.” (P2, male, 17 years, SHS 2)
Social support	“It is my grandmother who mostly reminds me of taking my medicine, or I sometimes remember by myself to take my medicine.” (P1, male, 15 years, JHS 3)
	“It is my grandparent who always reminds me to take my medicine. And she doesn't punish me when I miss taking my medication.” (P14, male, 15 years, JHS 3)
	“My family is also aware, so they sometimes check with me in the evenings if I have taken that day's dose yet, which provides an added reminder.” (P3, female, 14 years, JHS 1)
	“My mother also reminds me sometimes to take my medicine.” (P13, male, 14 years, JHS 1)
	“My family is also aware and they double-check with me sometimes if I have taken that day's dose.” (P12, male, 18 years, SHS 3)
Activity reminders	“For my medication, I take medicine after eating supper. I make sure I take it immediately because if I wait and get hungry, I will not get any food again to take with it.” (P4, male, 17 years, SHS 2)
	“As a Muslim, our evening prayers are at 7 pm, so I take my medicine each day after eating before the prayers.” (P14, male, 15 years, JHS 3)
	“After dinner each night, I take a few minutes to relax, and that is my cue to take my medication. Having that regimen has really solidified the habit.” (P12, male, 18 years, SHS 3)
	“We go to prep at 7 pm. When going to prep I will always take the required pill and put it in a plastic container and then put it in my school bag and take it to prep.” (P11, female, 18 years, SHS 3)
Technology (phone or watch alarm)	“I have set a daily alarm on my phone at 7 pm. At 7 pm, I take medicine after eating, and I take it by one pill daily.” (P5, male, 14 years, JHS 2)
	“I have an alarm set on my phone for 6:30 pm every day to remind me to take my pill after eating supper.” (P7, female, 18 years, tertiary year 1)
	“I have set an alarm on my phone to remind me each time at 8 pm to take my medicine.” (P19, male, 18 years, SHS 3)
	“I use my watch to know the time when it is on me, to know the time to take my medicine, and I take my medicine immediately after realizing that it is time.” (P13, male, 14 years, JHS 1)
	“The motivation for good health helps me remember to take my medication. Also I have a personal watch that I use to watch the time.” (P15, male, 16 years, SHS 1)

The quotations in Table 3 show that remembering to take ART was rarely left to chance, and that adolescents typically layered several methods rather than relying on any one. Under the social support sub-theme, adolescents leaned heavily on family members, and the specific relative varied with household composition in a way that is visible when the quotations are read against Table 1. The two participants living with a grandmother, P1 and P14, both named her as their principal reminder, while those living with parents described parental or general family checking. Where the relative knew the adolescent's status, they served as a dependable second layer of accountability, and the quality of that relationship mattered as much as the reminder itself: P14 noted specifically that his grandmother did not punish him when he missed a dose, indicating that a non-punitive relational stance made the reminder easier to accept. The youngest participants, P3 and P13, both aged 14, described family reminders as central rather than supplementary, whereas older participants such as P12 described family checking as a secondary layer over their own established routine, suggesting a developmental shift from external prompting toward self-regulation.

Under the activity reminders sub-theme, adolescents tied medication to fixed daily cues, most often the evening meal, deliberately taking the pill the moment food was available so that hunger later in the evening would not leave them without anything to take it with. As Table 3 shows, the anchoring cue was not always food. P14 anchored his dose to the timing of Islamic evening prayers, and P12 described a post-dinner period of rest as the trigger. Boarding students adapted the principle to institutional timetables, with P11 using evening preparatory study as the reference point and carrying a decanted dose to prep. Incorporating ART into an existing routine in this way made adherence a settled part of daily life rather than a separate task to be recalled.

Under the technology sub-theme, adolescents with access to a phone or a watch used alarms set to a fixed time, with reported dosing times clustering in the evening between 6:30 pm and 8 pm. Participants valued the personalisation and reliability these tools offered, and several described the alarm as the mechanism that established the habit initially, after which the routine itself took over. Watches served the same function for those without phones, as P13 and P15 described. As the opening quotations in Table 3 signal, the underlying driver for adopting any of these methods was a recognized vulnerability to forgetting amid the competing demands of school, compounded in some cases by hunger. The strategies therefore represent the practical behavioral skills adolescents devised to manage that vulnerability.

### 3.4 Theme two: stigma and concerns about privacy

Stigma and concern about privacy emerged strongly across the discussions, capturing the persistent anxiety adolescents felt about being identified as living with HIV. This theme had two sub-themes: maintaining privacy regarding medication, and concerns about HIV public education campaigns. Representative quotations are presented in Table 4.

**Table 4:** Representative quotations for Theme Two: stigma and concerns about privacy.

Sub-theme	Representative quotations
Maintaining privacy regarding medication	“I do not let anyone see me taking my medication. I always put the medicine in a different container before putting it in the drawer. When I need to take the medication somewhere, like when I am traveling, I transfer only the doses I will need into an unmarked pill case or container to maintain privacy.” (P6, male, 17 years, SHS 3)
	“In the early days of my diagnosis, I used to miss taking my medicine because I had just gone to SHS boarding, and my colleagues kept asking why I always took medicine and what that medicine was for. It was a great challenge because I did not know what to say; I only had to miss doses, especially when people are around.” (P4, male, 17 years, SHS 2)
	“My friends always come for items from my locker. So, when I put the medicine in a different container, they will not be able to determine the kind of medicine and the kind of illness that I have.” (P11, female, 18 years, SHS 3)
	“I have a particular bag that I put my medicine in. Immediately it is time, I straight away take my medication. I don't let anyone see it. The only persons who see it are my father and mother who bring me to the hospital.” (P2, male, 17 years, SHS 2)
	“I don't let anyone see me taking my medication, because of the questions that may arise such as what type of medicine am I taking and it is for what illness.” (P15, male, 16 years, SHS 1)
	“My friends used to ask me about my medication but I tell them it is a drug I take, and I just take it and I don't let people see me taking the drugs.” (P9, male, 19 years, tertiary year 2)
	“Firstly, I don't tell anyone about my illness. And my mother also told me not to tell anyone.” (P13, male, 14 years, JHS 1)
	“Upon my education on the illness, I was told that it is not a good thing to tell a lot of people or friends about your problem. Because of this I don't let anyone know that I have this illness.” (P2, male, 17 years, SHS 2)
	“My mum only informed my house mistress about my medication, but not the kind of medication.” (P18, female, 15 years, SHS 1)
Concerns about public education campaigns	“One concern I have is about the advertisements on TV that clearly show the bottles of ARV medication. Even though they are meant to educate, I find it too revealing of the type of medication I am taking. Sometimes I change the container to deter my friends and extended family members from knowing that I am taking this medication.” (P7, female, 18 years, tertiary year 1)
	“I used to keep my medication bottles in the refrigerator, but after seeing an advertisement about ARVs, I started hiding them instead. Now I keep the bottles in an opaque container in my wardrobe where others can't easily access them.” (P9, male, 19 years, tertiary year 2)
	“I feel some campaigns are actually too open in revealing personal medical information by clearly displaying the specific antiretroviral drug bottles and packaging. It feels like an invasion of privacy.” (P12, male, 18 years, SHS 3)
	“I felt very sad, afraid and stigmatized initially. Based on inaccurate portrayals of the disease in movies, I thought being HIV-positive meant I would certainly die soon.” (P7, female, 18 years, tertiary year 1)
	“Even though I contracted it from my mother at birth, I still felt a sense of shame and stigma about my condition. I was so worried about people judging me or treating me differently if they found out, because from what they show on TV, everyone with HIV will die.” (P8, female, 16 years, SHS 2)

Table 4 demonstrates how deeply the fear of disclosure shaped adolescents' day-to-day handling of their treatment, and the number of independent accounts makes clear that concealment was a normative practice rather than an idiosyncratic one. Nine participants across all three groups described some form of concealment. Under the privacy sub-theme, decanting medication into unmarked containers was the most common practice, alongside keeping medicines out of sight, timing doses to moments when no one was watching, and carrying only the required number of pills when traveling.

Reading the quotations against Table 1 shows that school setting intensified these pressures in a patterned way. The concealment accounts came disproportionately from boarding students, who described lockers as semi-public spaces routinely accessed by friends, making original packaging a specific hazard. P18, a 15-year-old boarding student living with her mother, described a partial disclosure strategy in which her mother told the house mistress that her daughter took medication while withholding what the medication was for, preserving institutional support without conceding the diagnosis. Day students, by contrast, more often described concealment within the household or from extended family rather than from institutional peers.

The accounts also revealed the cost of these efforts. As Table 4 shows, the pressure of peers' questions in boarding school had, at least early on, led P4 to miss doses rather than risk exposure, showing that privacy protection and adherence can come into direct tension. Several participants also described being instructed by parents or by health education not to tell anyone, with P13 and P2 attributing their secrecy directly to instruction from a mother and from clinical education respectively. Concealment was therefore reinforced by both family and clinical guidance rather than being solely self-generated.

Under the public education sub-theme, adolescents voiced a striking and recurrent concern that national HIV campaigns, by displaying recognizable ARV packaging on television, risked exposing the very young people they were meant to protect. This was not a single participant's view: it recurred across accounts from participants in different groups and at different educational levels, and in one case an advertisement prompted a direct behavioral change, with P9 moving his medication from the refrigerator to a concealed opaque container in a wardrobe. Alongside this, participants recalled the shame and fear that accompanied diagnosis, frequently traceable to frightening and inaccurate portrayals of HIV in films and on television that equated the condition with imminent death. Notably, both P7 and P8 attributed their initial belief that HIV meant imminent death to media portrayals rather than to clinical information. The theme therefore reveals a difficult balance, in which messaging intended to raise awareness can inadvertently heighten the disclosure anxieties that drive concealment.

### 3.5 Theme three: emotional and psychological impact

The emotional and psychological impact of living with HIV was a prominent concern, reflecting the deep feelings adolescents navigate while managing a lifelong condition. The discussions surfaced one central sub-theme, acceptance and coping through counseling, alongside a set of residual concerns that counseling had not resolved. Representative quotations are presented in Table 5.

**Table 5:** Representative quotations for Theme Three: emotional and psychological impact.

Sub-theme	Representative quotations
Acceptance and coping through counseling	“It all started in SHS 1, my first time in boarding school. I was getting sick frequently, and after lab investigations the doctor told me. The psychological and emotional struggle was very difficult at first after learning I was HIV-positive. My parents told me never to tell anyone, not even my best friend. I later told myself that it was not the end of the world. The nurses also supported me with counseling at every visit.” (P9, male, 19 years, tertiary year 2)
	“The counseling services helped a lot in coming to terms with it and understanding that HIV is nothing to be ashamed of if properly managed. But it took me a long time to accept it.” (P10, male, 15 years, SHS 1)
	“When I realized that I have this illness in SHS 2, I became sad. I had a goal of becoming a midwife, but because of this illness, I felt I would not be able to pursue that dream without potentially infecting someone. So I had to change my goals. I received counseling, and it has empowered me to take control of managing this condition in a healthy, responsible way without viewing it as something to feel ashamed about.” (P11, female, 18 years, SHS 3)
	“The counseling services helped me a lot in coming to terms with the reality that HIV is simply a chronic condition that can be managed properly. It's nothing to be ashamed of.” (P12, male, 18 years, SHS 3)
	“It was very difficult at first when I learned I had HIV, even though I contracted it from my mother at birth. I felt ashamed and worried people would judge me. But the counseling and education I received helped me understand it's nothing to be ashamed of if I take responsibility for my health.” (P7, female, 18 years, tertiary year 1)
	“I distanced myself from friends because I was scared of infecting them. It was only through the HIV education sessions that I learned the reality, that I could live a long, normal life by consistently taking the medication to suppress my viral load.” (P9, male, 19 years, tertiary year 2)
Residual and unresolved concerns	“The option of only taking the medicine always with no end is of my most concern. I asked when I will have to stop taking the medicine but I was not told.” (P13, male, 14 years, JHS 1)
	“At times, though I take my medicine, I feel like there is no change. When I come for check-up upon taking the medicine, I realize that my viral load rises rather than falling.” (P9, male, 19 years, tertiary year 2)
	“I don't get anybody to talk to when I have any concern regarding the illness or medication, but I am able to talk to a health professional when it is time for my visitation to the health facility.” (P15, male, 16 years, SHS 1)

The quotations in Table 5 trace an emotional journey that begins, for many adolescents, in distress and gradually moves toward acceptance. Diagnosis was commonly accompanied by sadness, fear, denial, and self-stigma, and for some it forced a painful revision of life goals, as in the case of P11, who set aside her ambition to become a midwife because she feared transmitting the virus. P9 described withdrawing from friendships out of a mistaken fear of transmitting HIV through ordinary contact, illustrating how informational deficits and emotional distress compounded one another.

Reading these accounts against the disclosure ages in Table 1 reveals a pattern worth noting. The most vivid and emotionally detailed accounts of adjustment came from participants disclosed to during adolescence rather than childhood. P9 and P11, disclosed to in senior high school years one and two respectively, both narrated a discrete before-and-after moment with a specific location and a specific emotional aftermath, whereas participants disclosed to in childhood tended to describe their status as a longstanding fact rather than as an event. This suggests that the timing of disclosure shapes not only whether adolescents know their status but the kind of psychological work required of them, and that those disclosed to later may need more intensive and more recent support.

Across these accounts, counseling appeared as the decisive mechanism through which acceptance was reached, and it was named independently by participants in all three groups and across the educational range. Adolescents described counseling at clinic visits as a safe space in which they could process difficult emotions without being judged, reframe HIV as a manageable chronic condition rather than a death sentence, and rebuild a sense of control and self-worth. The accounts suggest that this emotional progression is tied to better self-management, since adolescents who had reached acceptance described being more proactive about their health and more consistent in their adherence.

Table 5 also documents concerns that counseling had not resolved, and these are analytically important because they qualify the otherwise positive account. P13, the youngest voice in this theme, remained troubled by the indefinite duration of treatment and reported that his question about when he might stop had gone unanswered, a gap that is notable given that he had known his status since childhood and might therefore have been assumed to have received adequate explanation. P9 described the discouragement of adhering while observing his viral load rise rather than fall, an experience that connects emotional wellbeing directly to the adequacy of clinical explanation. P15 reported having no one to talk to between clinic appointments. Continued counseling thus served not only to repair the initial shock of diagnosis but to maintain psychological stability, and these residual concerns indicate precisely where that support was still falling short.

### 3.6 Theme four: motivation and commitment to treatment

Despite the challenges they described, adolescents displayed strong motivation and commitment to their treatment. This commitment was sustained by two sub-themes: personal goals and ambitions, and a desire for good health. Representative quotations are presented in Table 6.

**Table 6:** Representative quotations for Theme Four: motivation and commitment to treatment.

Sub-theme	Representative quotations
Personal goals and ambitions	“Staying strong and achieving my dreams motivates me.” (P7, female, 18 years, tertiary year 1)
	“That is what I wanted for myself too, the opportunity to pursue my ambitions without being hindered by complications from this condition.” (P12, male, 18 years, SHS 3)
	“I saw examples of people my age achieving their dreams and goals by staying committed to their medication. That's what I wanted for myself.” (P16, female, 17 years, SHS 2)
	“I encourage myself to take the medicine. This is because I have realized that I am a poor orphan, so if I don't take the medication and the illness becomes severe, nobody will be there to help me. This is what I use to encourage myself in taking my medication always.” (P2, male, 17 years, SHS 2)
	“I saw an advertisement on TV about adults who are HIV-positive but have an undetected viral load by taking their medication seriously. This is my motivation to adhere to my treatment.” (P9, male, 19 years, tertiary year 2)
Desire for good health	“I take my medicine on time to be able to be in good health to be able to go to school and study very well; I do not want to miss classes because of sickness.” (P11, female, 18 years, SHS 3)
	“I used to fall sick often before the medication. But after the medication, that has stopped. I used to have ringworm all over my hair, which was very shameful. So now if I want to prevent those small illnesses, I must take my medication daily.” (P14, male, 15 years, JHS 3)
	“I take my medication always in order to help me achieve low viral load.” (P15, male, 16 years, SHS 1)
	“My knowledge and understanding encourage me to take my medication always without missing any.” (P13, male, 14 years, JHS 1)
	“I have been told that if I take my medication always, I will not encounter opportunistic infections. Because of this I take my medicine always without missing any.” (P23, male, 16 years, SHS 1)
	“My experience of falling drastically ill that year is a harsh reminder of what can happen if I don't adhere to my treatment. I don't ever want to go through that again.” (P9, male, 19 years, tertiary year 2)

Table 6 shows that adolescents framed adherence as instrumental to the futures they wanted for themselves. Under the personal goals sub-theme, educational, professional, and personal aspirations functioned as powerful, forward-looking motivators, with adolescents describing daily pill-taking as the means by which they would remain well enough to pursue their ambitions. Adherence, in this framing, was about far more than maintaining a clinical parameter; it was about safeguarding the possibility of a full and productive life. This orientation was most articulate among the older participants in senior high and tertiary education, which is consistent with the developmental emergence of long-term goal-setting.

Notably, several participants located their motivation in observed exemplars, whether peers of their own age living well on treatment, as P16 described, or adults featured in televised accounts of achieving undetectable viral loads, as P9 described. This indicates that visible role models functioned as a motivational resource in their own right. P2, one of the two orphaned participants, articulated a starker version of the same logic, reasoning that because no one else would care for him if he became seriously ill, adherence was a form of self-protection. His account, read against his household circumstances in Table 1, illustrates how precarious family support can convert into an intensified rather than a diminished sense of personal responsibility.

Under the desire for good health sub-theme, adolescents drew on direct, embodied experience of the difference treatment had made. As Table 6 shows, several contrasted frequent illness before treatment with stability afterwards, and the visible, socially embarrassing nature of some pre-treatment infections gave the motivation an additional and immediate force, as in P14's account of visible skin infection. The most vivid instance came from P9, who had experienced a treatment interruption lasting about a year after his supply ran out while traveling, during which he fell repeatedly ill and lost substantial weight; the memory of that period operated for him as a continuing deterrent against missing doses. Others expressed motivation in more explicitly informational terms, with P13, P15, and P23 citing knowledge of viral load suppression and of protection against opportunistic infection as the reason for daily adherence. That these informational accounts came from among the younger participants suggests that clinical education had established an accurate rationale for treatment even where the aspirational framing of the older participants had not yet developed. Taken together, the two sub-themes show motivation drawing on an aspirational orientation toward the future, a concrete appreciation of the present benefits of staying well, and an accurate understanding of what treatment does.

## 4 Discussion

This study set out to understand how adolescents living with HIV in the Ashanti Region of Ghana care for themselves on ART, and what sustains their adherence over time. The four themes generated together portray adolescents as active, resourceful managers of their own treatment, and they point to clear, implementable opportunities for services to reinforce what young people already do.

### 4.1 Mapping the themes onto the IMB model

The IMB model holds that adherence information and adherence motivation exert their effects largely through adherence-related behavioral skills (13, 14). Mapping the four themes onto these components clarifies both what the model explains well in this setting and where it requires supplementation. The mapping is summarized in Table 7.

**Table 7:** Mapping of generated themes onto Information-Motivation-Behavioral Skills model components.

Theme	Primary IMB component	Secondary component	How the theme operates within the model
Theme One: ART reminder strategies	Behavioral skills	Motivation	Family prompts, routine cues, and alarms are the observable behavioral skills through which information and motivation are converted into daily pill-taking; family reminders also supply external motivation, most visibly among the youngest participants
Theme Two: Stigma and concerns about privacy	Moderator acting on all three components	Information	Stigma suppresses information-seeking, erodes social motivation, and forces the substitution of concealment skills for adherence skills; operates largely outside the individual and constrains the model's internal pathways
Theme Three: Emotional and psychological impact	Motivation	Information	Acceptance reached through counseling establishes the affective precondition for durable motivation; counseling simultaneously corrects inaccurate information about prognosis and transmission
Theme Four: Motivation and commitment to treatment	Motivation	Information	Personal goals supply intrinsic motivation, while embodied experience of health gains and accurate knowledge of viral suppression supply the informational grounding that makes motivation durable

As Table 7 indicates, Themes One and Four sit squarely within the model as originally specified. Theme One is the behavioral-skills component made visible in everyday practice, and Theme Four is the motivation component expressed in the language of adolescent aspiration. Theme Three occupies a position the model accommodates but does not foreground: emotional acceptance functions as a precondition for motivation rather than as a separate construct, and our data suggest that where acceptance was absent, information and motivation alone were insufficient to produce consistent behavior.

The age composition of our sample adds a developmental observation to this mapping. Among the youngest participants, adherence appeared to rest more heavily on the informational component and on externally supplied behavioral skills, with P13 attributing his adherence to knowledge and understanding and P3 and P13 both centring family prompting. Among the oldest, adherence rested more on internalized motivation grounded in personal goals, with behavioral skills having become habitual and largely self-sustaining. This suggests that the relative weight of the IMB components shifts across adolescence, and that adherence support should shift accordingly, emphasizing caregiver-mediated skills and accurate information in early adolescence and goal-oriented motivational work in later adolescence.

Theme Two is the component that fits least comfortably, and this is theoretically informative. Stigma did not act on any single element of the model but on all three simultaneously. It suppressed information-seeking, because adolescents avoided conversations that might expose their status; it eroded motivation, by cutting adolescents off from the social support that reinforces adherence; and, most consequentially, it forced adolescents to develop and prioritize a competing set of behavioral skills oriented toward concealment rather than toward adherence. The boarding-school accounts show these two skill sets in direct conflict, with concealment prevailing and doses being missed. This suggests that the IMB model, while a sound organizing framework for individual-level determinants, benefits from being embedded within a socio-ecological perspective when applied in high-stigma settings, since the model's internal pathways are themselves constrained by social and structural forces the model does not itself specify. This integration is reflected in the conceptual basis presented in Figure 1.

### 4.2 Reminder strategies as adolescent-engineered behavioral skills

The prominence of family reminders mirrors a literature identifying family support and cohesion as central facilitators of adherence among ALHIV in sub-Saharan Africa. Studies in southern Uganda have shown that social support and family cohesion are positively associated with adherence and with adherence self-efficacy among HIV-positive adolescents (32, 33), and social protection analyses in South Africa have demonstrated that supportive home environments improve adolescent ART adherence (34). The Ghanaian adolescents in this study were effectively operationalising that support themselves, recruiting grandmothers, parents, and other relatives as reminder partners.

Two features of our data extend this literature, and both are visible only because participant household composition was documented. First, grandmothers featured as principal reminder partners for precisely those participants who lived with them, reflecting the caregiving structures common where parents have died, which suggests that adherence support should be designed around whoever actually occupies the caregiving role rather than assuming a parent. Second, the relational quality of the reminder mattered, with P14 emphasizing that his grandmother did not punish missed doses. This carries a practical implication for implementation: where a trusted adult already knows the adolescent's status, services can formalize that person's role within the care plan as a named treatment supporter, and can coach them in non-punitive prompting rather than leaving the arrangement informal and unguided.

The use of daily routines reflects the principle of habit formation, which qualitative work in Kampala has identified as a key facilitator of durable adherence among people living with HIV (35). Anchoring a pill to an existing, reliable cue reduces the cognitive load of remembering and protects against the busy, irregular schedules adolescents repeatedly cited. Our data show the cue itself to be culturally and institutionally variable, encompassing the evening meal, Islamic evening prayers, and boarding-school preparatory study, which indicates that adherence counseling should help each adolescent identify a cue that is genuinely fixed in their own daily life rather than prescribing a standard one.

The reliance on phone and watch alarms is consistent with the digital-adherence literature. A systematic review and meta-analysis focused specifically on adolescents found that mobile phone text-message reminders can improve ART adherence in this age group, although the authors noted the limited and heterogeneous evidence base (36), and earlier reviews of electronic reminder devices reported modest benefits for medication adherence more generally (37). Implementation studies of text-message interventions among adolescents in nearby Ethiopia have examined the practical fidelity challenges of delivering such reminders at scale (38), and peer-led mobile health approaches have shown feasibility and acceptability for youth (39). Our findings suggest that Ghanaian adolescents are already willing adopters of these tools, which strengthens the case for integrating low-cost, confidential reminder supports into routine care. Two cautions follow from the data. Reminder tools were often described as scaffolding that established a habit which then became self-sustaining, implying that the goal of digital support should be habit formation rather than indefinite prompting. And phone access was not universal across the sample, with watch-based and routine-based cues serving the same function for those without a personal handset, which represents a more equitable design starting point than phone-dependent interventions.

### 4.3 Stigma, privacy, and the unintended effects of public messaging

The pervasiveness of stigma and the elaborate privacy work adolescents undertook align closely with the regional evidence. Perceived stigma and fear of unintended disclosure have repeatedly been identified as core barriers to adherence among adolescents with perinatal HIV, including a qualitative study in Botswana documenting concealment behaviors strikingly similar to those reported here (40), and HIV-related stigma has been linked directly to non-adherence (41). Internalized stigma and discrimination have been shown to compromise retention and adherence through psychosocial pathways including isolation and depression (42, 43), and stigma has been associated with mental-health difficulties among ALHIV in Malawi (44). That every participant in our sample had perinatally acquired infection makes the Botswana comparison particularly apt, since both samples faced the distinctive situation of managing a condition acquired before memory and disclosed by others.

School environments emerged in our data as the specific site where these pressures concentrated, a finding consistent with work documenting school-level barriers to adherence in Kenya and the particular difficulties of boarding secondary school environments in Uganda (45–47). This is where our participant characteristics are most illuminating: with thirteen of twenty-four participants in boarding accommodation, the concentration of concealment accounts among boarders is not an artifact of a few unusual voices but a pattern across the majority of the sample. The decanting of pills into unmarked containers, the treatment of shared lockers as hazards, and the episodes of missed doses to avoid peer questions all show adolescents trading adherence against anonymity. The partial-disclosure strategy described by P18, whose mother informed the house mistress that medication was required without revealing the diagnosis, is an instructive adaptation that secured institutional accommodation while limiting exposure, and it suggests a model that schools and clinics could formalize jointly.

A distinctive contribution of this study is the clarity with which adolescents articulated concern about HIV public education campaigns. Participants valued awareness in principle but objected that campaigns displaying recognizable ARV packaging on national television threatened to expose their status. The effect was behavioral and not merely attitudinal: P9 relocated and concealed his medication directly in response to an advertisement. This insight is underexplored in the literature and indicates that well-intentioned communication can carry an unintended disclosure cost for the people it depicts. It argues for co-designing campaign content with young people living with HIV so that messaging educates the public without compromising the privacy of those in treatment. The repeated references to frightening film and television portrayals that equated HIV with imminent death further suggest that correcting these narratives, and replacing them with accurate accounts of HIV as a manageable chronic condition, should be an explicit objective of public messaging. Notably, P9 cited precisely such an accurate portrayal, an advertisement featuring adults who had achieved undetectable viral loads, as a source of motivation, which demonstrates that the problem lies in how campaigns are designed rather than in public messaging as such.

### 4.4 Emotional impact and the central role of counseling

The emotional trajectory our participants described, moving from sadness, fear, and self-stigma at diagnosis toward acceptance, is well recognized in the literature on adolescent psychosocial adjustment to HIV, with qualitative work from Uganda, Botswana, and Namibia documenting the difficult emotional terrain of growing up with the condition (19, 48, 49). The strong, repeated identification of counseling as the mechanism of acceptance is consistent with the meta-analytic finding that counseling and education are principal facilitators of adherence among adolescents in sub-Saharan Africa (4). Enhanced adherence counseling has been shown to support young people with suspected treatment failure in Kenya (50), and realist and scoping reviews have clarified how psychosocial interventions improve adherence and viral load among adolescents and young people (51, 52).

Our documentation of age at disclosure permits an observation not available from the quotations alone. The participants who narrated the most acute emotional adjustment were those disclosed to during adolescence rather than childhood, and this aligns with cohort evidence showing that disclosure rates rise steeply with age and that a substantial minority of perinatally infected adolescents learn their status only in mid-adolescence (24, 25). The implication for services is that counseling intensity should be calibrated to time since disclosure rather than to chronological age alone, since a 19-year-old disclosed to at sixteen may be at an earlier point in the adjustment process than a 15-year-old who has known since childhood.

The link our participants drew between emotional acceptance and more consistent adherence resonates with evidence that self-acceptance and emotion regulation relate to treatment-adherence self-efficacy among adolescents with HIV (53), and with cohort and meta-analytic findings that mental health and psychiatric morbidity are closely intertwined with adherence and viral suppression (54, 55). Integrating mental health care into HIV services for adolescents is therefore not a peripheral addition but a direct adherence intervention (56).

Our data also identify where counseling was falling short, and these gaps are the most actionable part of this theme. Participants reported unresolved distress about the indefinite duration of treatment, discouragement at viral load results that did not improve despite adherence, and an absence of anyone to consult between scheduled visits. Each points to a specific service response: explicit and repeated explanation of lifelong therapy framed in terms of chronic disease management, structured clinical explanation when viral load results are discouraging so that adolescents do not interpret them as personal failure, and some form of between-visit contact such as a peer or counselor point of access. That the lifelong-treatment concern was raised by a 14-year-old disclosed to in childhood is a reminder that early disclosure does not guarantee adequate ongoing explanation. For Ghanaian services, the broader implication is that counseling should be resourced as a routine, repeated, and developmentally tailored element of every adolescent visit rather than a one-off event at diagnosis.

### 4.5 Motivation, aspiration, and commitment

The motivational accounts our participants offered represent the motivation component of the IMB model and are echoed across qualitative studies of adolescents and youth. A study in Kenya found that resilience and a sense of future possibility were important to engagement in HIV care (57), applications of the IMB model among youth have shown how motivation and behavioral skills together support adherence (58), and work on supporting adolescents to develop competencies for independent HIV management highlights the role of forward-looking goals in the transition to self-management (59).

Our findings add three nuances. First, motivation was not only aspirational but anchored in direct bodily experience of the benefits of treatment and, in one case, of the consequences of its interruption. Second, exemplars mattered: adolescents cited peers of their own age and adults portrayed as achieving undetectable viral loads as concrete evidence that the future they wanted was attainable. Third, the register of motivation varied with age across our sample, with the youngest participants expressing motivation in informational terms grounded in what they had been told about viral suppression and opportunistic infection, and the oldest expressing it in aspirational terms grounded in educational and professional goals. Services can harness all three registers by helping adolescents articulate specific personal goals, by reinforcing through accurate education the tangible health gains that adherence delivers, and by making positive exemplars visible through peer mentorship. This aspirational framing also offers a constructive counter-narrative to the fatalistic media portrayals discussed above.

### 4.6 Convergence with the quantitative strand

Reading the two strands of the parent study together strengthens the interpretation of both. The quantitative strand recorded 92% self-reported adherence but could not explain how such a level was achieved in a context of pervasive stigma. The qualitative themes supply that explanation: adherence was actively engineered through layered reminder systems, sustained by family involvement, and motivated by concrete aspirations. In IMB terms, the quantitative finding that adherence information and motivation were associated with behavioral skills is given observable content by the specific skills documented here.

The strands also illuminate one another where they appear to diverge. The quantitative analysis found only a weak association between behavioral skills and adherence, which is counterintuitive given the prominence of practical strategies in the narratives. The qualitative data suggest a resolution: because reminder strategies were near-universal in this population, they varied little across individuals and therefore had limited capacity to explain variance statistically, even though they were causally central to the behavior. Similarly, the finding that higher educational level predicted lower adherence is difficult to interpret from survey data alone, but the qualitative accounts point toward a mechanism. Thirteen of our twenty-four participants were in boarding accommodation, overwhelmingly at senior high and tertiary level, and it was precisely these participants who described the surveillance pressures, shared lockers, and peer questioning that led to concealment-driven missed doses. The educational gradient in the quantitative data may therefore reflect not education itself but the residential and institutional arrangements that accompany progression through the Ghanaian school system. This convergence illustrates the value of the parallel design and supports the case for interventions targeted at the boarding school environment specifically.

### 4.7 Implications for implementation and adolescent-friendly services

Read as a whole, the findings make a consistent implementation argument: the most acceptable and feasible adherence supports for Ghanaian adolescents are extensions of strategies they have already adopted. Formalizing trusted-adult reminders with non-punitive coaching and with the caregiver identified from the adolescent's actual household rather than an assumed one, helping each adolescent select a genuinely fixed routine cue, embedding confidential digital reminders where phone access permits while providing watch-based or routine-based alternatives where it does not, protecting privacy in both clinical encounters and public messaging, establishing partial-disclosure protocols with boarding schools, and resourcing repeated counseling calibrated to time since disclosure would each build on demonstrated adolescent practice rather than impose external solutions.

These directions are consistent with evidence that differentiated and adolescent-friendly service models, including youth-friendly clinics and teen-club approaches, can support retention and adherence in sub-Saharan Africa (60, 61), and with broader reviews of interventions to enhance HIV medication adherence and improve adolescent HIV care outcomes (17, 62). They are also relevant in the Ghanaian policy context, where pooled adherence among adolescents and young adults sits well below the level required for reliable viral suppression (7), and where mixed-method facility studies have called for service-level responses to both patient and system barriers (11, 63). Over the longer term, emerging options such as long-acting antiretrovirals may ease some of the privacy and routine burdens adolescents described by reducing the frequency of visible pill-taking (64), although their accessibility in settings such as Ghana remains to be established.

### 4.8 Strengths and limitations

A strength of this study is its focus on adolescent agency, surfacing the affirmative care strategies young people use rather than cataloging barriers alone, and its grounding in a theoretically informed qualitative process with independent peer coding, member checking, prospective assessment of saturation, and a documented audit trail. Full participant characteristics are reported, allowing readers to judge the patterning of accounts by age, sex, schooling arrangement, household composition, and time since disclosure. Conducting discussions in Asante Twi with independent translation verification allowed participants to express themselves naturally. Requiring documented disclosure before enrolment protected participants and ensures that the accounts come from adolescents with established awareness of their status. Embedding the strand within a convergent parallel design permitted triangulation with quantitative findings from the same sites.

Several limitations should be borne in mind, and the first concerns social desirability, which we regard as the most consequential threat to the validity of these findings. Adherence was self-reported, in a group setting, to a research team wearing nursing uniforms and introduced as part of the health team. Each of these features independently raises the likelihood that participants overstated their adherence, and together they make the strongly adherence-affirming tone of many accounts difficult to interpret at face value. Adolescents may have been particularly reluctant to describe non-adherence in front of peers from their own clinic, given that admitting missed doses in that setting risks both social judgement and, as participants perceived it, disappointing clinical staff. The risk is not evenly distributed across the sample: the three 14-year-old participants sat in groups containing 18- and 19-year-olds, and younger participants in mixed-age groups are more likely to defer to an emerging consensus than to contradict it. The practical implication is that the *prevalence* of adherence suggested by these accounts, and by the parallel 92% self-report, should be treated with considerable caution, and that the *strategies* described are the more reliable contribution of this study. Some counterweight exists in the data, since participants did disclose missed doses, concealment-driven omissions, and a year-long treatment interruption, which indicates that the normalizing introduction and non-judgemental facilitation had some effect. Nonetheless, non-adherence is likely to be under-represented, and experiences that deviate most sharply from the adherent norm are the least likely to have surfaced.

Relatedly, the focus group format, while well suited to eliciting shared and normative practices, is not the appropriate vehicle for the most private experiences. Perinatally infected adolescents are often reluctant to discuss their status in company, and the group setting may have exerted a conformity pressure in which early speakers established an adherent narrative that later speakers matched. Groups were also mixed-sex, and although the care strategies under discussion were not sex-specific, we cannot exclude the possibility that some experiences, particularly those touching on relationships or sexual health, went unvoiced for that reason. Individual interviews, or a sequential design combining groups with follow-up interviews, would likely have surfaced a fuller account.

Further limitations concern scope and method. The study was conducted in three facilities in a single region, so transferability to other regions or to community and rural settings should be considered with care, although the thick description provided supports reasoned transfer. With one focus group per facility stratum, code saturation was reached but meaning saturation for facility-specific issues is less securely established, and additional groups per stratum would have strengthened this. Eligibility was restricted to adolescents aged 14 to 19, so the experiences of early adolescents aged 10 to 13, who face different disclosure and autonomy dynamics and who were included in the quantitative census, are not represented here. All participants had perinatally acquired infection, so the findings may not transfer to adolescents with behaviourally acquired HIV, whose disclosure circumstances and treatment histories differ substantially. The sample was drawn from adolescents engaged in care, so the perspectives of those who had disengaged are absent, and these are likely the adolescents facing the greatest difficulty. Household circumstances were not recorded for two participants, a minor gap in the characterization of the sample. Formal back-translation was not performed, and although independent verification against the original audio was undertaken, some nuance may have been lost in translation. Finally, as a qualitative study, this work describes and interprets practices rather than quantifying their prevalence or testing their effectiveness.

### 4.9 Future directions

Future work should test whether formalizing the adolescent-engineered strategies identified here, in particular trusted-adult reminder partnerships, confidential routine and digital cues, school-based partial-disclosure protocols, and repeated counseling calibrated to time since disclosure, improves adherence and viral suppression in Ghanaian adolescents, ideally through implementation-oriented designs that measure acceptability, feasibility, and fidelity alongside objective clinical outcomes such as viral load, which would circumvent the self-report limitations discussed above. Research co-designed with adolescents on the disclosure effects of HIV public education campaigns is warranted, as is work that includes adolescents who have disengaged from care, that extends to early adolescents and to those with behaviourally acquired infection, that reaches community and rural contexts, and that uses individual interview methods to access the experiences group settings cannot.

## 5 Conclusion

Adolescents living with HIV in the Ashanti Region of Ghana are not passive recipients of treatment but active architects of their own adherence. They recruit whichever trusted adult heads their household as a reminder partner, anchor their dosing to fixed daily cues ranging from the evening meal to evening prayers and school preparatory study, use phone and watch alarms, conceal their medication in unmarked containers to protect their privacy, and draw on counseling and personal ambition to sustain long-term commitment. The balance among these resources shifts across adolescence, from externally supplied prompting and accurate information in the younger years toward internalized, goal-driven motivation in the older ones. Participants also voiced a clear and actionable concern that HIV public education campaigns can inadvertently expose their status, and identified specific gaps in counseling around the indefinite duration of treatment. For services seeking to improve adolescent adherence and advance progress toward viral suppression targets, the most promising path is to formalize and strengthen the strategies adolescents already use, to protect privacy in the clinic, the boarding school, and public messaging alike, and to make repeated, developmentally appropriate counseling a routine part of care. Centring adolescent agency in this way offers a practical and humane foundation for adolescent-friendly ART services in Ghana and comparable settings.

## Data Availability

The original contributions presented in the study are included in the article/Supplementary material. The full focus group transcripts are not publicly available because they contain sensitive information that could compromise the privacy of adolescents living with HIV; de-identified excerpts are available from the corresponding author on reasonable request and subject to ethical approval. Further inquiries can be directed to the corresponding author.

## Supplementary material

Supplementary Data Sheet 1 Analytic audit trail for the qualitative strand: de-identification protocol (Section A), codebook (Table S1), de-identified excerpts mapped to codes (Tables S2 and S3), and coded thematic summaries with analytic memos (Table S4).
