# Supplementary material for: ART adherence care strategies among adolescents living with HIV in Ghana's Ashanti Region: a qualitative study
Source: Front Public Health. 2026 Sep 16;14:1915477. doi: 10.3389/fpubh.2026.1915477 (PMC13624123; doi:10.3389/fpubh.2026.1915477)
Supplement: Supplementary file 1 [file Supplementary_file_1.docx]

**Supplementary Material**

**De-identified qualitative excerpts and coded thematic summaries supporting the manuscript**

**ART adherence care strategies among adolescents living with HIV in Ghana's Ashanti Region: a qualitative study**

This data sheet documents the analytic audit trail for the qualitative strand: the de-identification protocol, the full codebook, the de-identified excerpts mapped to codes, and the coded thematic summaries. It is provided to support transparency, dependability, and confirmability, and to allow readers to trace each reported theme back to the underlying data.

**Section A. De-identification protocol**

The following procedures were applied so that no participant can be identified from this file.

1. **Field-assigned codes.** During data collection, each participant was assigned a numeric session code and asked to state that code, rather than a name, before speaking. Names were never recorded. The numeric codes used below (for example, P01) are these field-assigned anonymized tags. The key linking codes to individuals is held securely by the research team and is not included in any supplementary file.
2. **Removal of direct identifiers.** Names of participants, relatives, schools, communities, and specific health facilities have been removed or replaced with generic terms in square brackets (for example, [boarding school], [the health facility]).
3. **Generalization of quasi-identifiers.** Rare relational or biographical details that could, in combination, identify a participant in a small community have been generalized. Where a detail was generalized, this is flagged with a bracketed note (for example, [sibling]). Exact ages reported in the main manuscript are retained for the validated excerpts in Table S2 for consistency with the published quotations; for any onward data release, age banding (14 to 15, 16 to 17, 18 to 19 years) is recommended.
4. **Translation.** Discussions were conducted in Asante Twi and transcribed and translated into English. All excerpts are presented in English translation. Minor non-substantive smoothing of translated grammar was applied only where needed for readability, without altering meaning. Ellipses (...) indicate omitted text; square brackets indicate clarifying insertions.
5. **Demographic detail.** Where a validated demographic descriptor (sex, age, education stage) was confirmed for a quoted participant in the source thesis, it is shown. Where only the field code is reliably known, demographic detail is withheld and marked accordingly.

**Section B. Codebook (Table S1)**

The codebook was developed inductively in NVivo against a shared coding frame, with independent peer coding and consensus resolution of discrepancies. Four primary themes and their sub-themes are reported in the manuscript. Five contextual (minor) codes did not rise to standalone themes but informed interpretation and triangulation, and are documented here for completeness.

**Table S1.** Codebook: themes, sub-themes, code identifiers, and operational definitions.

| **Theme** | **Sub-theme** | **Code ID** | **Code label** | **Operational definition** |
| --- | --- | --- | --- | --- |
| 1. ART reminder strategy | Social support | RS-FAM | Family reminder | A relative (for example, grandmother or parent) prompts the adolescent to take the dose |
|  | Social support | RS-CHK | Family verification | A relative checks or confirms that the dose has been taken, adding a second layer of accountability |
|  | Activity reminders | RS-MEAL | Meal-anchored dosing | The dose is tied to a daily meal, usually supper, so eating cues the medication |
|  | Activity reminders | RS-PRAYER | Prayer-anchored dosing | The dose is tied to a fixed daily religious routine (for example, evening prayers) |
|  | Technology | RS-ALARM | Device alarm | A phone or watch alarm set to a fixed time prompts the dose |
| 2. Stigma and concerns about privacy | Maintaining privacy | PR-CONT | Unmarked/decanted container | Pills are transferred into unlabelled containers or pill cases to disguise the medication |
|  | Maintaining privacy | PR-CONCEAL | Discreet dosing | The adolescent takes medication out of others' sight and avoids being observed |
|  | Maintaining privacy | PR-STORE | Concealed storage | Medication is stored out of sight and out of reach to limit discovery |
|  | Maintaining privacy | PR-MISS | Privacy-adherence tension | A dose is delayed or missed specifically to avoid exposure to peers or others |
|  | Public education campaigns | PE-ADVERT | Campaign packaging exposure | Concern that televised campaigns display recognizable ARV packaging and risk revealing status |
|  | Public education campaigns | PE-MEDIA | Inaccurate media portrayal | Fear or shame traced to film/TV portrayals equating HIV with imminent death |
|  | Public education campaigns | PE-SHAME | Blameless shame | Shame felt despite perinatal acquisition and absence of personal fault |
| 3. Emotional and psychological impact | Acceptance and coping | EM-DISTRESS | Initial distress | Sadness, fear, denial, or self-stigma at and after diagnosis |
|  | Acceptance and coping | EM-SECRECY | Instructed secrecy | Parental or guardian instruction to tell no one about the status |
|  | Acceptance and coping | EM-GOALS | Revised life goals | Diagnosis prompting reconsideration or change of personal aspirations |
|  | Acceptance and coping | EM-COUNSEL | Counselling as acceptance pathway | Counselling enabling acceptance, reframing of HIV as manageable, and empowerment |
| 4. Motivation and commitment to treatment | Personal goals and ambitions | MO-DREAM | Aspiration as motivator | Future educational, professional, or personal goals drive adherence |
|  | Desire for good health | MO-HEALTH | Health preservation | Staying well and avoiding illness motivates adherence |
|  | Desire for good health | MO-EXPERIENCE | Embodied benefit | Direct memory of pre-treatment illness and post-treatment improvement reinforces adherence |
| Contextual (minor) codes | (cross-cutting) | CC-INTERRUPT | Treatment interruption | A period off treatment driven by supply exhaustion or travel, with health consequences |
|  | (cross-cutting) | CC-ORPHAN | Orphanhood and self-reliance | Orphanhood or economic vulnerability shaping self-reliant motivation |
|  | (cross-cutting) | CC-SIDE | Tolerability issues | Side effects or administration difficulties (for example, dizziness on an empty stomach, large pills) |
|  | (cross-cutting) | CC-ACCESS | Future access concern | Worry about continued access to medication after a transition (for example, leaving school) |
|  | (cross-cutting) | CC-OI | Opportunistic infection prevention | Self-care practices to prevent infection (handwashing, nutrition, mosquito avoidance, rest) |

**Section C. De-identified excerpts mapped to codes**

**Table S2. Validated representative excerpts (as cited in the manuscript)**

These excerpts carry confirmed demographic descriptors and correspond to the quotations presented in Tables 3 to 6 of the manuscript.

**Table S2.** Validated de-identified excerpts by code, with confirmed participant descriptors.

| **Code ID** | **Participant descriptor** | **Excerpt** |
| --- | --- | --- |
| RS-FAM | Male, 15 y, JHS3 | "It is my grandmother who mostly reminds me of taking my medicine, or I sometimes remember by myself to take my medicine." |
| RS-CHK | Female, 14 y, JHS1 | "My family is also aware, so they sometimes check with me in the evenings if I have taken that day's dose yet, which provides an added reminder." |
| RS-MEAL | Male, 17 y, SHS2 | "For my medication, I take medicine after eating supper. I make sure I take it immediately because if I wait and get hungry, I will not get any food again to take with it." |
| RS-ALARM | Male, 14 y, JHS2 | "I have set a daily alarm on my phone at 7 pm. At 7 pm, I take medicine after eating, and I take it by one pill daily." |
| RS-ALARM (motivating concern) | Male, 16 y, SHS1 | "The nurses have been telling me that taking my medications on time is important, but I sometimes forget if I am too busy with school activities. I needed something that will remind me every day." |
| PR-CONT / PR-CONCEAL | Male, 17 y, SHS3 | "I do not let anyone see me taking my medication. I always put the medicine in a different container before putting it in the drawer. When I need to take the medication somewhere, like when I am travelling, I transfer only the doses I will need into an unmarked pill case or container to maintain privacy." |
| PR-MISS | Male, 17 y, SHS2 | "In the early days of my diagnosis, I used to miss taking my medicine because I had just gone to [boarding school], and my colleagues kept asking why I always took medicine and what that medicine was for. It was a great challenge because I did not know what to say; I only had to miss doses, especially when people are around." |
| PE-ADVERT | Female, 18 y, Tertiary (year 1) | "One concern I have is about the advertisements on TV that clearly show the bottles of ARV medication. Even though they are meant to educate, I find it too revealing of the type of medication I am taking. Sometimes I change the container to deter my friends and extended family members from knowing that I am taking this medication." |
| PE-MEDIA | Female, 18 y, Tertiary (year 1) | "I felt very sad, afraid and stigmatised initially. Based on inaccurate portrayals of the disease in movies, I thought being HIV-positive meant I would certainly die soon." |
| PE-SHAME / PE-MEDIA | Female, 16 y, SHS2 | "Even though I contracted it from my mother at birth, I still felt a sense of shame and stigma about my condition. I was so worried about people judging me or treating me differently if they found out, because from what they show on TV, everyone with HIV will die." |
| EM-DISTRESS / EM-SECRECY / EM-COUNSEL | Male, 19 y, Tertiary (year 2) | "It all started in [SHS 1], my first time in [boarding school]. I was getting sick frequently... after lab investigations, the doctor told me. The psychological and emotional struggle was very difficult at first after learning I was HIV-positive. My parents told me never to tell anyone, not even my best friend. I later told myself that it was not the end of the world. The nurses also supported me with counselling at every visit." |
| EM-COUNSEL | Male, 15 y, SHS1 | "The counselling services helped a lot in coming to terms with it and understanding that HIV is nothing to be ashamed of if properly managed. But it took me a long time to accept it." |
| EM-GOALS / EM-COUNSEL | Female, 18 y, SHS3 | "When I realised that I have this illness in [SHS 2], I became sad. I had a goal of becoming a midwife, but because of this illness, I felt I would not be able to pursue that dream without potentially infecting someone. So I had to change my goals. I received counselling, and it has empowered me to take control of managing this condition in a healthy, responsible way without viewing it as something to feel ashamed about." |
| MO-DREAM | Female, 18 y, Tertiary (year 1) | "Staying strong and achieving my dreams motivates me." |
| MO-DREAM | Male, 18 y, SHS3 | "That is what I wanted for myself too, the opportunity to pursue my ambitions without being hindered by complications from this condition." |
| MO-HEALTH | Female, 18 y, SHS3 | "I take my medicine on time to be able to be in good health to be able to go to school and study very well; I do not want to miss classes because of sickness." |
| MO-EXPERIENCE / MO-HEALTH | Male, 15 y, JHS3 | "I used to fall sick often before the medication. But after the medication, that has stopped. I used to have ringworm all over my hair, which was very shameful. So now if I want to prevent those small illnesses, I must take my medication daily." |

**Table S3. Additional supporting excerpts from the focus group transcript**

These excerpts informed the codes and support triangulation but were not quoted in the manuscript. They are tagged with field participant codes; demographic detail beyond what was reliably recorded is withheld for anonymity, and identifying specifics have been generalized.

**Table S3.** Additional de-identified supporting excerpts by code.

| **Code ID** | **Participant tag** | **Excerpt** |
| --- | --- | --- |
| RS-FAM | P01 | "My grandmother helps remind me, or sometimes I remember on my own." |
| RS-PRAYER | P05 | "I take my medicine at 7 pm each day after eating and before our evening prayers. This routine helps me remember." |
| RS-ALARM | P09 | "I have an alarm set for 6:30 pm to remind me. As soon as the alarm goes off, I take my pill right away after eating supper." |
| RS-MEAL | P02 | "If there is no food, I still take the medicine and eat later." |
| PR-CONT | P03 | "I always put the medicine in a different container before going to [school]. My friends always come for items from my locker, so this way they cannot tell what kind of medicine it is." |
| PR-STORE | P09 | "I used to keep my medication in [a cold-storage place at home], but after seeing an advertisement I started hiding them instead. Now I keep the bottles in an opaque container in my [wardrobe] where others cannot easily access them." |
| PR-CONCEAL | P07 | "My friends used to ask me about my medication, but I did not let people see me taking the drugs. I just told them it was a drug I take." |
| PE-ADVERT | P05 | "There is an advertisement on TV that shows the exact container of the medicine I am taking. My roommate saw it and started asking questions about what kind of medicine I was on. I had to mislead her to protect my privacy." |
| EM-DISTRESS | P01 | "When I realised that I have HIV, I wept. I had not been involved in any [risk behaviour], so I wondered how I contracted it. I have a [sibling], but I am the only one with the virus." |
| EM-COUNSEL | P13 | "The counselling and education have been invaluable, especially in those initial struggles. It has empowered me to manage this condition responsibly and pursue my goals without feeling ashamed." |
| MO-EXPERIENCE / CC-INTERRUPT | P09 | "There was a period of about [a long time] when I stopped taking my medication because the supply I had brought while travelling ran out. My health declined badly and I kept falling sick, until I returned and restarted treatment and started improving again." |
| MO-HEALTH / CC-ORPHAN | P02 | "I encourage myself to take the medicine because I have realised that I am [without parents]. If I do not take the medication and the illness becomes severe, nobody will be there to help me." |
| MO-DREAM | P07 | "I saw an advertisement on TV about adults who are HIV-positive but who have an undetectable viral load by taking their medication seriously. This is my motivation." |
| CC-SIDE | P03 | "The large pills sometimes get stuck in my throat. I buy additional meals before taking my medicine to avoid dizziness." |
| CC-ACCESS | P08 | "My main concern is making sure I can always access my medication, even after I finish [school] and move somewhere else." |
| CC-OI | P01 | "I always wash my hands when I come back from playing so I do not get sick." |
| CC-OI | P06 | "I do not stay outside for long in the evening to prevent mosquito bites, I wash my hands frequently, and I eat well." |

**Section D. Coded thematic summaries (Table S4)**

For each theme, the table records the contributing codes, an analytic memo describing the pattern across participants, the spread of participants contributing (described qualitatively, consistent with the thesis and avoiding precise counts that the data do not support), and the link to the IMB construct used as a sensitising framework.

**Table S4.** Coded thematic summary with analytic memos.

| **Theme** | **Contributing codes** | **Analytic memo (pattern across participants)** | **Participant spread** | **IMB construct** |
| --- | --- | --- | --- | --- |
| 1. ART reminder strategy | RS-FAM, RS-CHK, RS-MEAL, RS-PRAYER, RS-ALARM | Adolescents rarely left dosing to memory alone. They layered reminders, drawing first on relatives who prompted and verified doses, then on fixed daily cues such as supper or evening prayers, and, where a device was available, on phone or watch alarms set to a constant time. The methods were complementary rather than mutually exclusive, and the underlying driver was an explicit awareness of forgetting amid school demands. | The majority; reminder use was near-universal, with family support most common among younger adolescents and device alarms more common among those with phone access | Behavioural skills |
| 2. Stigma and concerns about privacy | PR-CONT, PR-CONCEAL, PR-STORE, PR-MISS, PE-ADVERT, PE-MEDIA, PE-SHAME | Concealment was a routine, practised behaviour: decanting pills into unmarked containers, storing medicine out of sight, and dosing only when unobserved. Privacy work sometimes competed directly with adherence, with at least one account of missed doses to avoid peer questions. A distinctive and recurrent concern was that televised campaigns displaying recognizable ARV packaging threatened disclosure, prompting some to change containers. Fear and shame were frequently traced to inaccurate media portrayals of HIV. | Most participants reported privacy practices; campaign concern and media-driven fear were voiced across school stages, including tertiary | Information (corrective need) and behavioural skills (concealment) |
| 3. Emotional and psychological impact | EM-DISTRESS, EM-SECRECY, EM-GOALS, EM-COUNSEL | Diagnosis was commonly met with sadness, fear, denial, and self-stigma, sometimes compounded by instructions to keep the status secret and, for some, a painful revision of life goals. Counselling at clinic visits emerged consistently as the mechanism through which adolescents reached acceptance, reframed HIV as manageable, and regained a sense of control. Acceptance was associated in participants' accounts with more proactive self-management. | Many participants; counselling was described as pivotal across the group, with acceptance more consolidated among older adolescents | Motivation (personal) and information |
| 4. Motivation and commitment to treatment | MO-DREAM, MO-HEALTH, MO-EXPERIENCE | Commitment drew on two registers. The first was aspirational, with educational, professional, and personal goals framing adherence as the means to a full life. The second was embodied, grounded in direct memory of frequent pre-treatment illness contrasted with stability afterwards, which made the cost of non-adherence vivid and immediate. | The majority expressed strong commitment; aspirational framing and embodied benefit appeared across school stages | Motivation (personal) |
| Contextual (minor) codes | CC-INTERRUPT, CC-ORPHAN, CC-SIDE, CC-ACCESS, CC-OI | These codes did not form standalone themes but recurred and shaped interpretation. A cautionary account of a long treatment interruption from supply exhaustion illustrated the consequences of non-adherence; orphanhood and economic vulnerability shaped self-reliant motivation; tolerability issues such as dizziness on an empty stomach and large pills influenced timing and food practices; future access worries surfaced around transitions; and a range of self-care practices to prevent opportunistic infection accompanied medication-taking. | A subset of participants; each contextual code was raised by one or several participants | Cross-cutting; primarily behavioural skills and motivation |
