# Supplementary material for: ART adherence care strategies among adolescents living with HIV in Ghana's Ashanti Region: a qualitative study
Source: Front Public Health. 2026 Sep 16;14:1915477. doi: 10.3389/fpubh.2026.1915477 (PMC13624123; doi:10.3389/fpubh.2026.1915477)
Supplement: Supplementary file 2 [file Supplementary_file_2.docx]

**UNIVERSITY OF CAPE COAST**

**COLLEGE OF EDUCATION STUDIES**

**DEPARTMENT OF HEALTH, PHYSICAL EDUCATION AND RECREATION**

**QUESTIONNAIRE FOR ADOLESCENTS**

**Dear Valued Respondents,**

My name is Doris Hagan, a PhD student at the Department of Physical Education and Recreation, University of Cape Coast studying Health Promotion with specialization in Maternal and Child Health. I am undertaking a research study on; **“Determinants of adherence to antiretroviral treatment among adolescents living with HIV/AIDS in the Ashanti Region of Ghana.”** The aim of this study is to determine factors influencing adherence to ART among adolescents living with HIV/AIDS in the Ashanti Region of Ghana.” Please, you are required to complete a 54-item questionnaire which may take 30 to 45minutes of your time. All other adolescents who visit the health selected facilities in the Ashanti Region will be required to complete this questionnaire and all responses will be analyzes as a group. Your responses will assist in planning ways to help other adolescents who must take pills on a difficult schedule. Please do your best to answer all the questions. If you do not wish to answer a question, please draw a line through it. If you do not know how to answer a question, ask the researcher or assistant to help. Your identity is kept anonymous, and your participation is entirely voluntary. You also have the right to stop answering the questionnaire at any time or withdraw from the study. If you understand your role as a respondent to this study and agree to participate, please append your signature or thumb print in the space provided below.

Signature ........................................... date ................................

For any information, please contact my supervisors Prof. Charles Domfeh (0544756528) or Dr. Thomas Hormenu (0244213465). You may also contact me (Doris Hagan) on 0244372710 or [doris.hagan@stu.ucc.edu.gh](mailto:doris.hagan@stu.ucc.edu.gh)

Thank you for helping in this important study.

**SECTION A: Socio-Demographic details**

**Please mark [√ ] in the box matching respondent’s choice on the statements below, write your response where applicable.**

**Code number ..............................**

1. **Gender**: a. Male	[	]

b. Female	[	]

2. **Age** ......................... years
3. **Indicate the level of your education**
a. No formal education	[	]
b. Primary	[	]
c. JHS	[	]
d. SHS	[	]
e. Vocation	[	]
f. Tertiary	[	]
4. **What is your Relationship status? (this applies to teen mothers)**
a. Never married	[	]
b. Married	[	]
c. Divorced	[	]
5. **Who are you Living with?**
a. Alone	[	]
b. Partner	[	]
c. Parent	[	]
d. Family member	[	]
e. Others, please specify ........................
6. **How long have you been on ARVs?** ........................ years
7. **What is your Religion?**
a. Christian	[	]
b. Muslim	[	]
c. Traditional	[	]
d. Others, please specify …………….
8. **How will you classify your family economic status?**
a. Low	[	]
b. Middle	[	]
c. High	[	]
9. **What is the Source of your infection?**
a. Vertical	[	]
b. Horizontal	[	]
c. Unknown	[	]
10. **How long has it been since you were diagnosed with HIV infection? ………..**
11. **Do you have someone who support you to take your ARTs?**
a. Yes	[	]
b. No	[	]
12. **What is your latest Viral Load results?** ......................................................
13. **When was it taken?** ……………………..
14. **How long have you been started on antiretroviral treatment?**
a. 6 – 11months	[	]
b. 1 – 2years	[	]
c. More than 2years	[	]

**SECTION B: INSTRUCTIONS: This page is to be completed by the patient and study researcher together.**

15. **You are currently taking the following drugs at the frequency and doses listed.**

| Drug Name/Dose | # Pills Each Time  (Pills Each Dose) | # Times Per Day  (Doses Per Day) |
| --- | --- | --- |
|  |  |  |
|  |  |  |
|  |  |  |
|  |  |  |
|  |  |  |
|  |  |  |

**The next section of the questionnaire asks about your medications that you took over the last four (4) days.**

Most people with chronic illness have many pills to take at different times during the day.

Many people find it hard to always remember their pills:

- Some people get busy and forget to carry their pills with them.
- Some people find it hard to take their pills according to all the instructions, such as “with meals” or “on an empty stomach,” “every 8 hours,” “with plenty of fluids.”
- Some people decide to skip doses to avoid side effects or to just not be taking pills that day.

We need to understand how you are really doing with your pills. Please tell us what you are **actually** doing. Don’t worry about telling us that you don’t take all your pills. We need to know what is really happening, not what you think we “want to hear.”

**The next section of the questionnaire asks about the study medications that you may have missed taking over the last four days. Please complete the following table by filling in the boxes below.**

16. If you took only a portion of a dose on one or more of these days, please report the dose(s) as being **missed.**

| **Step 1**  Names of your drugs | **HOW MANY DOSES DID YOU MISS** | | | |
| --- | --- | --- | --- | --- |
|  | **Step 2**  Yesterday | **Step 3**  Day before yesterday (2days ago) | **Step 4**  3 Days ago | **Step 5**  4 days ago |
|  |  |  |  |  |
|  |  |  |  |  |
|  |  |  |  |  |
|  |  |  |  |  |
|  |  |  |  |  |
|  |  |  |  |  |
|  |  |  |  |  |

**The following questions pertain to the study regimen above**

**If you took only a portion of a dose on one or more of these days, please report the dose(s) as being missed.**

17. **During the past 4days, on how many days have you missed taking all your doses? Please tick as applicable.**
a. None	[	]
b. One day	[	]
c. Two days	[	]
d. Three days	[	]
e. Four	[	]
18. **Most of your medications need to be on a schedule, such as “2times a day” or “3times a day” or every 8 hours.” How closely did you follow your specific schedule over the last four days?**
a. Never	[	]
b. Some of the times	[	]
c. About half of the time	[	]
d. Most of the time	[	]
e. All of the time	[	]
19. **Do any of your medications have special instructions, such as “take with food” or “on an empty stomach” or “with plenty of fluids**?”
a. Yes	[	]
b. No	[	]

**If yes, how often did you follow those special instructions over the last four days?**

a. Never	[	]
b. Some of the time	[	]
c. About half of the time	[	]
d. Most of the time	[	]
e. All of the time	[	]
20. **Some people find that they forget to take their pills on the weekend days. Did you miss any of your medications last weekend – last Saturday or Sunday?**
a. Yes	[	]
b. No	[	]
21. **When was the last time you missed any of your medications? Please check one.**
a. Within the past week	[	]
b. 1-2 weeks ago	[	]
c. 2-4 weeks ago	[	]
d. 1-3months ago	[	]
e. More than 3months ago	[	]
f. Never skip medication or

Not applicable	[	]

*If you* ***Never*** *skip medications, please go to* ***question number 23*** *otherwise, please continue by answering the next set of questions.*

22. **People may miss taking their medications for various reasons. Here is a list of possible reasons why you may miss taking your medications. How often have you missed taking your medications because you: (Circle one response for each question)**

| **Reasons** | **Never** | **Rarely** | **Sometimes** | **Often** |
| --- | --- | --- | --- | --- |
| a. Were away from home? | 0 | 1 | 2 | 3 |
| b. Were busy with other things? | 0 | 1 | 2 | 3 |
| c. Simply forgot? |  |  |  |  |
| d. Had too many pills to take? | 0 | 1 | 2 | 3 |
| e. Wanted to avoid side effects? | 0 | 1 | 2 | 3 |
| f. Did not want others to notice you taking the medication? | 0 | 1 | 2 | 3 |
| g. Had a change in daily routine? | 0 | 1 | 2 | 3 |
| h. Felt like the drug was toxic/harmful? | 0 | 1 | 2 | 3 |
| i. Fell asleep/ slept through dose time? | 0 | 1 | 2 | 3 |
| j. Fell sick or ill? | 0 | 1 | 2 | 3 |
| k. Felt depressed/ overwhelmed? | 0 | 1 | 2 | 3 |
| l. Had problems taking meals at specified times (with meals, on empty stomach, etc.) | 0 | 1 | 2 | 3 |
| m. Ran out of pills? | 0 | 1 | 2 | 3 |
| n. Felt good/well? | 0 | 1 | 2 | 3 |

| 23. **The following questions ask about symptoms you might have had during the past four weeks. Please circle the number that best describes how much you have been bothered by each symptom.**     **I DO NOT HAVE**  **THIS SYMPTOMS**  **I HAVE THIS SYMPTOM AND ...** | | | | | |
| --- | --- | --- | --- | --- | --- |
|  | **It doesn’t**  **bother me** | | **It bothers me a little** | **It bothers me a lot** | **It bothers me terribly** |
| a. Fatigue or loss of energy? | 0 | 1 | 2 | 3 | 4 |
| b. Fevers, chills or sweats? | 0 | 1 | 2 | 3 | 4 |
| c. Feeling dizzy or lightheaded? | 0 | 1 | 2 | 3 | 4 |
| d. Pain, numbness or tingling in the hands or feet? | 0 | 1 | 2 | 3 | 4 |
| e. Trouble remembering? | 0 | 1 | 2 | 3 | 4 |
| f. Nausea or vomiting? | 0 | 1 | 2 | 3 | 4 |
| g. Diarrhoea or loose bowel movements? | 0 | 1 | 2 | 3 | 4 |
| h. Felt sad, down or depressed? | 0 | 1 | 2 | 3 | 4 |
| i. Felt nervous or anxious? | 0 | 1 | 2 | 3 | 4 |
| j. Difficulty falling or staying asleep? | 0 | 1 | 2 | 3 | 4 |
| k. Skin problems, such as rash, dryness or itching? | 0 | 1 | 2 | 3 | 4 |
| l. Cough or trouble catching your breath? | 0 | 1 | 2 | 3 | 4 |
| m. Headache? | 0 | 1 | 2 | 3 | 4 |
| n. Loss of appetite or a change in the taste of food? | 0 | 1 | 2 | 3 | 4 |
| o. Bloating, pain or gas in your stomach? | 0 | 1 | 2 | 3 | 4 |
| p. Muscle aches or joint pain? | 0 | 1 | 2 | 3 | 4 |
| q. Problems with having sex, such as loss of interest or lack of satisfaction? | 0 | 1 | 2 | 3 | 4 |
| r. Changes in the way your body looks, such as fat deposits or weight gain? | 0 | 1 | 2 | 3 | 4 |
| s. Problems with weight loss or wasting? | 0 | 1 | 2 | 3 | 4 |
| t. Hair loss or changes in the way your hair looks? | 0 | 1 | 2 | 3 | 4 |

**SECTION C: QUESTIONNAIRE ITEMS ON ADHERENCE INFORMATION, MOTIVATION AND BEHAVIORAL SKILLS.**

**INSTRUCTIONS: This page is to be completed by the patient and study researcher together. Please tick [√]**

| **Adherence Information** | **Yes** | **No** |
| --- | --- | --- |
| 24. Do you know how each of your current medications is supposed to be taken (for example whether or not to be taken with food, or other prescribed medications)? | **[ ]** | **[ ]** |
| 25. Do you know what to do if you miss a dose of any of my medications (for example, whether or not to take the pill (s) later)? | **[ ]** | **[ ]** |
| 26. Do you know skipping a few of your medications from time to time would really hurt your health? | **[ ]** | **[ ]** |
| 27. Do you know what the possible side effects of each of your medications are? | **[ ]** | **[ ]** |
| 28. Do you know that although you are feeling healthy, missing your HIV medications from time to time is not good? | **[ ]** | **[ ]** |
| 29. Do you know how each of your medications work in my body to fight the disease? | **[ ]** | **[ ]** |
| 30. Do you know that if you do not take your medications as prescribed, it may not work for you in the future? | **[ ]** | **[ ]** |
| 31. Do you believe that if you take you medications as prescribed, you will live longer? | **[ ]** | **[ ]** |
| 32. Do know your medications interact with alcohol and hard drugs? | **[ ]** | **[ ]** |

**INSTRUCTIONS: Please tick (√) as applicable to your views.**

| **Motivation** | **Strongly disagree** | **Disagree** | **Not sure** | **Agree** | **Strongly agree** |
| --- | --- | --- | --- | --- | --- |
| 33. I get frustrated taking my medications because I have to plan my life around them. | 1 | 2 | 3 | 4 | 5 |
| 34. I do not like taking my medications because they remind me of my condition | 1 | 2 | 3 | 4 | 5 |
| 35. I feel that my healthcare provider takes my needs into account when making recommendations about which medications to take | 1 | 2 | 3 | 4 | 5 |
| 36. Most people who are important to me who know my condition support me in taking my medication | 1 | 2 | 3 | 4 | 5 |
| 37. My health care provider does not give me enough support when it comes to taking my medications as prescribed. | 1 | 2 | 3 | 4 | 5 |
| 38. It frustrates me to think that I will have to take these medications every day for the rest of my life | 1 | 2 | 3 | 4 | 5 |
| 39. I am worried that the medications I have been prescribed will hurt my health | 1 | 2 | 3 | 4 | 5 |
| 40. It upsets me that the medications I have been prescribed can affect the way I look | 1 | 2 | 3 | 4 | 5 |
| 41. It upsets me that the medications I have been prescribed can cause side effects**.** | 1 | 2 | 3 | 4 | 5 |
|  |  |  |  |  |  |
| **Behavioural skills** | **Very hard** | **Hard** | **Normal** | **Easy** | **Very Easy** |
| 42. How hard or easy is it for you to stay informed about HIV treatment? | 1 | 2 | 3 | 4 | 5 |
| 43. How hard or easy is it for you to get the support you need from others for taking your medications (for example, from friends, family, doctor or pharmacist) | 1 | 2 | 3 | 4 | 5 |
| 44. How hard or easy is it for you to get your medication refills on time? | 1 | 2 | 3 | 4 | 5 |
| 45. How hard or easy is it for you to take your medications when you are wrapped up in what you are doing? | 1 | 2 | 3 | 4 | 5 |
| 46. How hard or easy is it for you to manage the side effects of your medications? | 1 | 2 | 3 | 4 | 5 |
| 47. How hard or easy is it for you to remember to take your medications? | 1 | 2 | 3 | 4 | 5 |
| 48. How hard or easy is it for you to take your medications because the pills are hard to swallow, taste bad, or make you sick to your stomach? | 1 | 2 | 3 | 4 | 5 |
| 49. How hard or easy is it for you to make your medications part of your daily life? | 1 | 2 | 3 | 4 | 5 |
| 50. How hard or easy is it for you to take your medications when your usual routine changes (for example, when you travel or when you go out with your friends)? | 1 | 2 | 3 | 4 | 5 |
| 51. How hard or easy is it for you to take your medications when you do not feel good emotionally (for example, when you are depressed, sad, angry, or stressed out)? | 1 | 2 | 3 | 4 | 5 |
| 52. How hard or easy is it for you to take your medications when you feel good physically and do not have any symptoms of your disease? | 1 | 2 | 3 | 4 | 5 |
| 53. How hard or easy is it for you to take your medications when you do NOT feel good physically? | 1 | 2 | 3 | 4 | 5 |
| 54. How hard or easy is it for you to talk to your health care provider about your HIV medications? | 1 | 2 | 3 | 4 | 5 |
