# Supplementary material for: ART adherence care strategies among adolescents living with HIV in Ghana's Ashanti Region: a qualitative study
Source: Front Public Health. 2026 Sep 16;14:1915477. doi: 10.3389/fpubh.2026.1915477 (PMC13624123; doi:10.3389/fpubh.2026.1915477)
Supplement: Supplementary file 3 [file Supplementary_file_3.docx]

**QUALITATIVE QUESTIONNAIRE**

**UNIVERSITY OF CAPE COAST
COLLEGE OF EDUCATION STUDIES**

**DEPARTMENT OF HEALTH, PHYSICAL EDUCATION AND RECREATION**

**FOCUS GROUP DISCUSSION GUIDE FOR ADOLESCENTS**

**Dear Adolescent,**

I am a PhD (Health Promotion) candidate who is conducting a research survey on the **“Determinants of adherence to antiretroviral treatment among adolescents living with HIV/AIDS in the Ashanti Region of Ghana.”** The aim of this study is; “To determine factors that influence adherence to ART among adolescents living with HIV/AIDS in the Ashanti Region of Ghana.” Please be informed that all responses you give in this survey are confidential and used for research purposes only. This discussion may take between 60 to 90 minutes of your time. Your participation in this study is completely voluntary and you are free to even stop answering the items if you deem it necessary. Thank you for your participation.

**Section A: Care strategies adopted by Adolescents in the care of HIV/AIDS:**

1. Can you explain to me in details and in turns, the strategies you have adopted to care for yourself?

**Prompts:**

i. What is your experience when it comes to taking your daily medications (ARTs)?
ii. What is your experience when it comes to protecting yourself from getting opportunistic infections?
iii. What is your experience when it comes to keeping your medication safe?
iv. What is your experience when it comes to following the treatment regimen?
v. Can you share your experience on how you handle challenges with caring for yourself as far as this condition is concerned?
vi. What has been the main thing that has helped you to adhere to your medication?
vii. What has been the main thing that has prevented you from adhering to your treatment?
     2. **Is there any other thing you will like to share aside what we have talked about?**

**Thank you very much for your time.**

**We may contact you again if we need further clarifications on your responses.**
